# Drug sensitivity profiling of 3D tumor tissue cultures in the pediatric precision oncology program INFORM

**DOI:** 10.1038/s41698-022-00335-y

**Published:** 2022-12-27

**Authors:** Heike Peterziel, Nora Jamaladdin, Dina ElHarouni, Xenia F. Gerloff, Sonja Herter, Petra Fiesel, Yannick Berker, Mirjam Blattner-Johnson, Kathrin Schramm, Barbara C. Jones, David Reuss, Laura Turunen, Aileen Friedenauer, Tim Holland-Letz, Martin Sill, Lena Weiser, Christopher Previti, Gnanaprakash Balasubramanian, Nicolas U. Gerber, Johannes Gojo, Caroline Hutter, Ingrid Øra, Olli Lohi, Antonis Kattamis, Bram de Wilde, Frank Westermann, Stephan Tippelt, Norbert Graf, Michaela Nathrath, Monika Sparber-Sauer, Astrid Sehested, Christof M. Kramm, Uta Dirksen, Olli Kallioniemi, Stefan M. Pfister, Cornelis M. van Tilburg, David T. W. Jones, Jani Saarela, Vilja Pietiäinen, Natalie Jäger, Matthias Schlesner, Annette Kopp-Schneider, Sina Oppermann, Till Milde, Olaf Witt, Ina Oehme

**Affiliations:** 1 Hopp Children’s Cancer Center Heidelberg (KiTZ), 69120 Heidelberg, Germany; 2 Clinical Cooperation Unit Pediatric Oncology, German Cancer Research Center (DKFZ) and German Cancer Consortium (DKTK), 69120 Heidelberg, Germany; 3 National Center for Tumor Diseases (NCT), 69120 Heidelberg, Germany; 4 Faculty of Biosciences, Heidelberg University, 69120 Heidelberg, Germany; 5 Bioinformatics and Omics Data Analytics, German Cancer Research Center (DKFZ), 69120 Heidelberg, Germany; 6 Division of Pediatric Neurooncology, German Cancer Research Center (DKFZ) and German Cancer Consortium (DKTK), 69120 Heidelberg, Germany; 7 Faculty of Mathematics and Computer Science, Heidelberg University, 69120 Heidelberg, Germany; 8 Clinical Cooperation Unit Neuropathology, German Cancer Research Center (DKFZ), German Consortium for Translational Cancer Research (DKTK), 69120 Heidelberg, Germany; 9 Division of Pediatric Glioma Research, German Cancer Research Center (DKFZ) and German Cancer Consortium (DKTK), 69120 Heidelberg, Germany; 10 Department of Pediatric Oncology, Hematology, Immunology and Pulmonology, Heidelberg University Hospital, 69120 Heidelberg, Germany; 11 Department Neuropathology at Institute of Pathology, Heidelberg University Hospital, 69120 Heidelberg, Germany; 12 Institute for Molecular Medicine Finland (FIMM), Helsinki Institute of Life Science (HiLIFE), University of Helsinki, 00014 Helsinki, Finland; 13 Division of Biostatistics, German Cancer Research Center (DKFZ), Heidelberg, Germany; 14 Core Facility Omics IT and Data Management (ODCF), German Cancer Research Center (DKFZ), 69120 Heidelberg, Germany; 15 Department of Oncology, University Children’s Hospital Zürich, CH-8032 Zürich, Switzerland; 16 Department of Pediatrics and Adolescent Medicine, Comprehensive Cancer Center and Comprehensive Center for Pediatrics, Medical University of Vienna, 1090 Vienna, Austria; 17 St. Anna Children’s Hospital, Department of Pediatrics and Adolescent Medicine, Medical University of Vienna, 1090 Vienna, Austria; 18 St. Anna Children’s Cancer Research Institute (CCRI), Vienna, Austria; 19 Children’s Hospital, Pediatric Oncology, Skåne University Hospital, Lund & Karolinska University Hospital, Stockholm, Sweden; 20 Faculty of Medicine and Health Technology, Tampere Center for Child Health Research, Tampere University, Tampere, Finland, and Tays Cancer Center, Tampere University Hospital, Tampere, Finland; 21 First Department of Pediatrics, National and Kapodistrian University of Athens, Athens, Greece; 22 Center for Medical Genetics, Ghent University, Ghent, Belgium; 23 Division of Neuroblastoma Genomics, German Cancer Research Center, Heidelberg, Germany; 24 Pediatrics III Pediatric Hematology, Oncology, Immunology, Cardiology, Pulmonology, West German Cancer Center; German Cancer Consortium (DKTK), University Hospital Essen, Essen, Germany; 25 Department of Pediatric Oncology, Saarland University Medical Center, 66421 Homburg, Germany; 26 Department of Pediatric Oncology, Klinikum Kassel, Kassel, Germany; 27 Department of Pediatrics and Children’s Cancer Research Center, Klinikum rechts der Isar, Technical University of Munich, Munich, Germany; 28 Klinikum der Landeshauptstadt Stuttgart gKAöR, Olgahospital, Stuttgart Cancer Center, Zentrum für Kinder-, Jugend- und Frauenmedizin, Pädiatrie 5 (Pädiatrische Onkologie, Hämatologie, Immunologie), Stuttgart, Germany; 29 University of Medicine Tübingen, Tübingen, Germany; 30 Department of Pediatrics and Adolescent Medicine, Rigshospitalet, Copenhagen Denmark; 31 Division of Pediatric Hematology and Oncology, University Medical Center Göttingen, Göttingen, Germany; 32 iCAN Digital Precision Cancer Medicine Flagship, University of Helsinki, Helsinki, FI-00014 Finland; 33 Biomedical Informatics, Data Mining and Data Analytics, Faculty of Applied Computer Science and Medical Faculty, University of Augsburg, Augsburg, Germany; 34 Present Address: Clinical Cooperation Unit Neuropathology, German Cancer Research Center (DKFZ), German Consortium for Translational Cancer Research (DKTK), 69120 Heidelberg, Germany

**Keywords:** Paediatric cancer, Translational research, High-throughput screening, Targeted therapies

## Abstract

The international precision oncology program INFORM enrolls relapsed/refractory pediatric cancer patients for comprehensive molecular analysis. We report a two-year pilot study implementing ex vivo drug sensitivity profiling (DSP) using a library of 75–78 clinically relevant drugs. We included 132 viable tumor samples from 35 pediatric oncology centers in seven countries. DSP was conducted on multicellular fresh tumor tissue spheroid cultures in 384-well plates with an overall mean processing time of three weeks. In 89 cases (67%), sufficient viable tissue was received; 69 (78%) passed internal quality controls. The DSP results matched the identified molecular targets, including BRAF, ALK, MET, and TP53 status. Drug vulnerabilities were identified in 80% of cases lacking actionable (very) high-evidence molecular events, adding value to the molecular data. Striking parallels between clinical courses and the DSP results were observed in selected patients. Overall, DSP in clinical real-time is feasible in international multicenter precision oncology programs.

## Introduction

High-risk and progressive or relapsed cancers in children remain a significant challenge in pediatric oncology, with progression-free and overall survival rates of 10% and 20% at two years, respectively, despite intense multimodal treatment approaches^1^. To address this unmet medical need, the INFORM pediatric precision oncology program (INdividualized Therapy FOr Relapsed Malignancies in Childhood) was established to provide a comprehensive molecular diagnostic pipeline for target identification, including low-coverage whole-genome sequencing (lcWGS), whole-exome sequencing (WES), RNA sequencing (RNA-Seq), 850k DNA methylation profiling and clinical follow-up. After an initial pilot phase^2^, INFORM has now been rolled out to an international, real-world registry including 13 countries and more than 100 pediatric oncology centers with over 2000 patients enrolled to date. In addition to MOSCATO^3^, ZERO^4^, Genomes for Kids (G4K)^5^ and MAPPYACT^6^, INFORM^1^ is one of the largest pediatric precision oncology programs worldwide. Importantly, we have recently reported on the outcome of the first cohort of 519 patients with at least two years of clinical follow-up, demonstrating a doubling of progression-free survival (PFS) times in a subgroup of patients receiving molecularly matched targeted treatment^1^. This group primarily consists of patients with tumors harboring very high evidence level targets, such as activating genetic alterations of *ALK*, *BRAF,* and *NTRK*. However, most patients (>90%) with lower evidence targets did not show improved PFS outcomes when receiving molecularly matched treatment. Thus, one of the study’s major conclusions is that pediatric precision oncology programs need to be further developed beyond identifying actionable molecular alterations through sequencing-based technologies. Future precision oncology studies may benefit from the introduction of complex biomarkers and functional drug sensitivity profiling (DSP)^1^, which has also been proposed by others^4,7–9^. To this end, we initiated DSP on viable fresh tumor tissue, long-term culture and PDX model development. Based on previous data on 3D versus 2D drug response^10–12^, conceptual considerations of tumor tissue heterogeneity and clinical feasibility aspects, we developed a patient-derived short-term ex vivo 3D fresh culture format for DSP.

We report on the INFORM personalized DSP pipeline under clinical real-world conditions. We identified drug sensitivities in a high proportion of pediatric cancer patients, including those lacking actionable genetic events, indicating the potential added value of DSP in the context of pediatric precision oncology platforms.

## Results

**Fig. 1:**
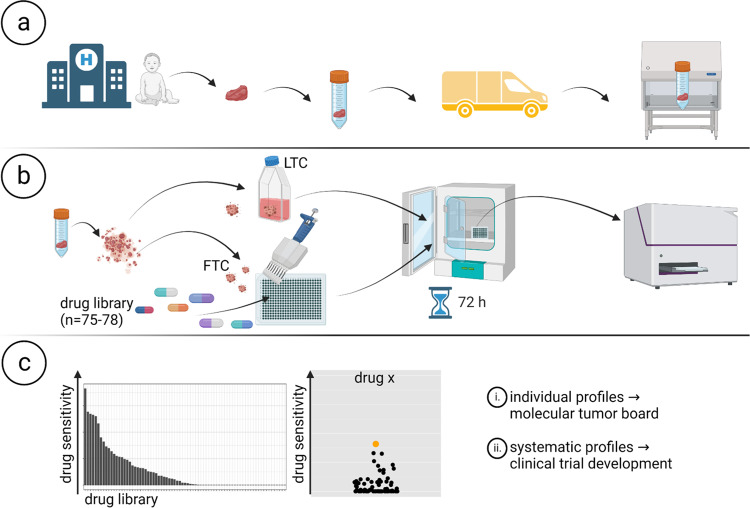
Workflow of INFORM personalized drug sensitivity profiling. Created with BioRender.com. **a** Sample collection and shipment. **b** Generation of patient-derived ex vivo fresh tissue spheroid cultures (FTCs) and treatment with a drug library. Readout: metabolic activity. **c** Data collection, analysis and preparation of drug sensitivity reports.

To add a functional component to our pediatric precision oncology program, INFORM, we initiated a personalized drug sensitivity and resistance profiling platform based on metabolic activity measurements complementing next-generation sequencing (NGS)-based target identification. The platform consists of three principal steps (Fig. 1): (a) collection and shipment of viable tumor tissue, (b) dissociation of tumor tissue, followed by generation and drug treatment of patient-derived ex vivo fresh tissue spheroid cultures (FTCs) in 384-well drug plates, and (c) data collection and determination of individual drug sensitivity and resistance profiles to be reported to the INFORM molecular tumor board. In parallel, long-term cultures (LTCs) and PDX models are being established for expanded drug testing approaches and clinical trial development.

### Collection and shipment of viable tumor tissue (a)

**Fig. 2:**
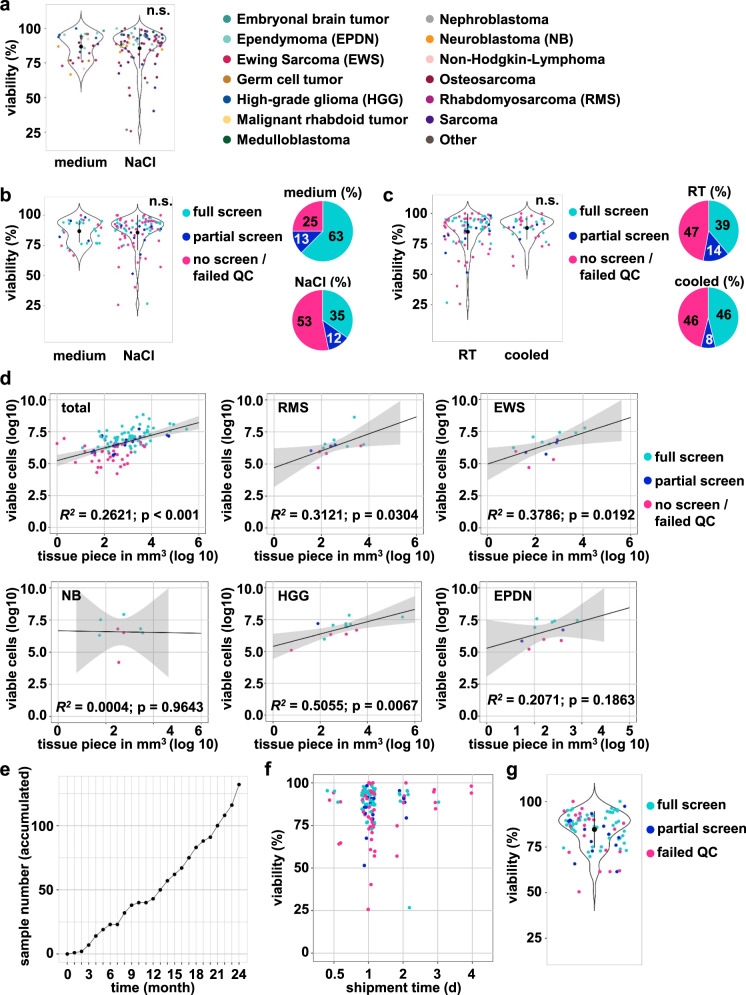
Assessment of shipment conditions and tissue viability. **a** Effect of sample shipment in 0.9% NaCl versus medium on sample viability, measured with automated viable and dead cell counting (ViCell or Cellometer). The color code on the right reflects the different tumor diagnoses. The black dots indicate the mean, error bars reflect SD. Students *t*-test (two-sided). **b** Violin plot: Effect of sample shipment in 0.9% NaCl versus cell culture medium on sample viability. Colors indicate screen type (full, meaning all three plates versus partial, meaning 1–2 plates versus no screen/screen that failed QC). The black dots indicate the mean, error bars reflect SD. Students *t*-test (two-sided). Pie diagrams: percentage of samples per screen type for samples shipped in 0.9% NaCl or medium. The color code in the middle reflects the screen type. **c** Violin plot: Effect of sample shipment at room temperature versus cooled on sample viability, with colors indicating the screening type. The black dots indicate the mean, error bars reflect SD. Students *t*-test (two-sided). Pie diagrams: percentage of samples per screening type for samples shipped at room temperature or code. The color code in the middle reflects the screen type. **d** Correlation plots calculated after log10 transformation comparing the volume of tissue piece to the viable cell number and the type of screen (full, meaning all three plates versus partial, meaning 1–2 plates versus no screen/screen (full or partial) that failed QC). The gray area represents the confidence intervals. Not all samples are displayed as volume or cell number data were not always available. Statistical method: Pearson correlation with R. **e** Accumulated incoming sample number of the two-year pilot phase (*n* = 132). **f** Effect of transport duration on sample viability, measured with automated viable and dead cell counting (ViCell or Cellometer). The color code reflects the tumor diagnoses as in panel **b**). **g** Viability at DSP seeding. The color code reflects the type of screen (full, meaning all three plates versus partial, meaning 1–2 plates versus screen that failed QC). The black dots indicate the mean, error bars reflect SD. EPDN ependymoma, EWS Ewing sarcoma, NB neuroblastoma, HGG high-grade glioma, RMS rhabdomyosarcoma. n.s.: not significant.

As a multicenter, multinational real-world precision oncology program, INFORM receives tumor material from 13 countries and over 100 pediatric oncology centers, many of which are nonuniversity centers with limited laboratory support. Thus, to keep the shipment conditions for viable tumor tissue as simple as possible, we allowed the shipment of tissue at room temperature or cooled overnight either in physiological sodium chloride solution or in any serum-free cell culture medium (e.g., RPMI, Neurobasal or other) (see “shipment protocol” Supplementary Note 1). We did not observe significant differences in the viability of incoming tissue shipped in 0.9% NaCl solution versus cell culture medium, although only samples shipped in NaCl (6/92) showed viability values <60% (Fig. 2a). Furthermore, screening success was higher if the sample was shipped in medium versus 0.9% NaCl solution (Fig. 2b). A comparison of the viability between samples shipped at room temperature versus sampled shipped cooled similarly revealed no significant difference in the samples, and shipment temperature did not affect the screening success rate (Fig. 2c). Although the study protocol requires a minimum tissue size of a “pea-sized tissue fragment”, incoming tissue piece sizes varied significantly depending on the extent of surgical resection. The average tissue volume was 3070 mm^3^, and 80% of all samples with a size ≥250 mm^3^ could be screened, while this was only possible in ~50% of all samples smaller than 250 mm^3^. However, in individual cases, successful screens could be performed on smaller tissue samples derived from stereotactic biopsies with sizes as low as 12 mm^3^. Notably, a correlation between tissue volume and total derived viable cell numbers and, hence, successful drug screening was observed for rhabdomyosarcomas, Ewing sarcomas and high-grade gliomas but not for neuroblastomas and ependymomas (Fig. 2d).

During the first two years (June 2019 to the end of May 2021) of the INFORM DSP pilot program, we received 132 viable tumor tissue samples from 122 patients (Fig. 2e) from 35 pediatric oncology sites in seven European countries (Germany, *n* = 21; Switzerland, *n* = 5; Austria, *n* = 2; Belgium, *n* = 2; Finland, *n* = 2; Sweden, *n* = 2; Greece, *n* = 1) mostly within 48 h after surgery (mean shipment time 1.2 days). We did not observe substantial differences in the average shipment time and yield of tumor cells for drug screens in Germany versus cases from other countries. Notably, a few samples in transit for as long as four days still demonstrated >90% viability. However, screening success was seemingly higher with a shipment time of fewer than 3 days (Fig. 2f). The average cell viability at seeding for DSP was 85%, with no major difference in the average viability between screens that passed quality control (QC; full or partial, 87% and 82%) or failed QC (81%), indicating that high viability upon seeding does not guarantee screening success. However, with one exception, all samples (*n* = 4) with viability <65% failed QC (Fig. 2g). Of note, from 89 drug screens (full or partial), 69 (78%) passed internal QC (Supplementary Fig. 1). Despite the large sample heterogeneity in terms of tumor diagnoses, a successful screen was performed for all diagnoses except for the two non-Hodgkin-lymphoma samples (Supplementary Fig. 2). Shipment conditions, screening success rates and QC scores for all diagnoses are summarized in Supplementary Data Table 1.

### Tissue dissociation and 3D tumor spheroid preculture (b)

**Fig. 3:**
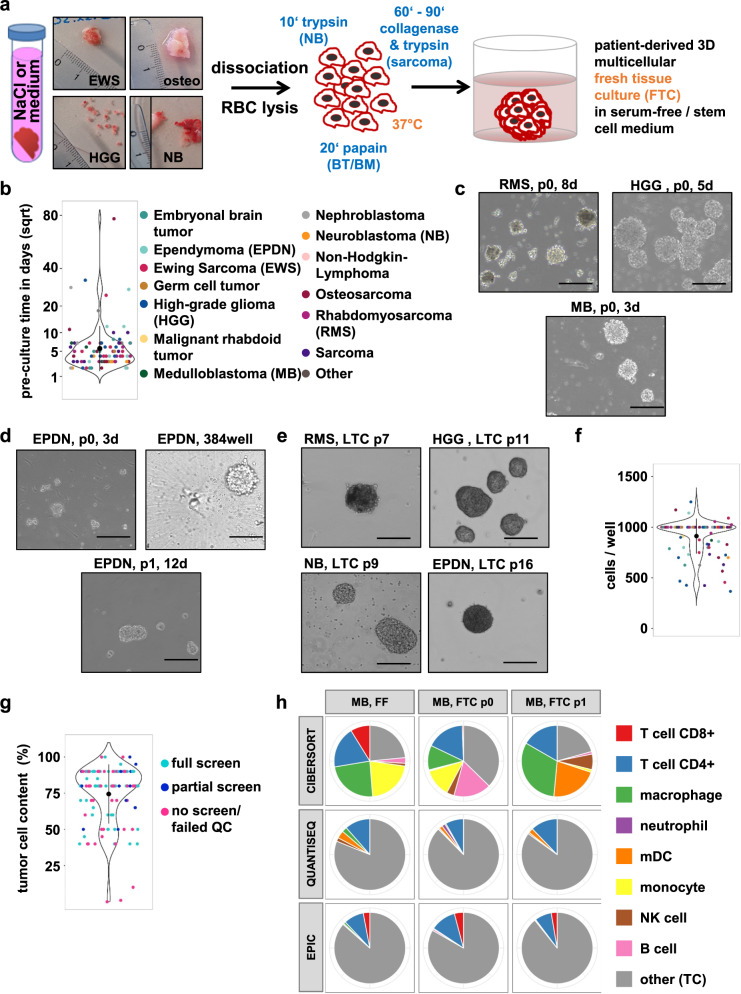
Dissociation of tumor tissue and characterization of the patient-derived 3D culture models. **a** Workflow of incoming tissue processing until the generation of patient-derived 3D multicellular fresh tissue culture (FTC). **b** Violin dot depicting the preculture time of screened samples in days; the *y*-axis is square-root transformed (sqrt) to better illustrate the distribution of all data points. The color code reflects the different tumor diagnoses (same as in **d**). The black dots indicate the mean, error bars reflect SD. **c** Bright field images (×10 magnification, cropped) for patient-derived 3D FTC precultures (d3, d5, d8) at passage 0. **d** Bright-field image (×10 magnification, cropped) for a patient-derived 3D culture from the same ependymoma (EPDN) sample at p0 (d3), at drug screen (384-well) and p1. **e** Bright-field images (×10 magnification, cropped) for patient-derived 3D long-term cultures (>p6, LTC). **f** Violin dot plot displaying the seeding cell number per well of the screened samples. The color code on the right reflects the different tumor diagnoses. The black dots indicate the mean, error bars reflect SD. **g** Violin dot plot illustrating the tumor cell content (in percent) of fresh frozen material accompanying the fresh tumor specimen submitted for DSP. The color code reflects the screening type (full, meaning all three plates versus partial, meaning 1–2 plates versus no screen/screen that failed quality control (QC)). The black dots indicate the mean, error bars reflect SD. **h** Immune cell type deconvolution results from the same medulloblastoma (MB) sample from FF (fresh frozen; original tumor), at p. 0 (directly after dissociation) and p. 1 (seeding time-point) with the most commonly used bulk RNA-seq deconvolution tools: CIBERSORT, QuantiSeq, and EPIC. TC tumor cell.

As we initially experienced significant challenges in trying to dissociate and preculture all incoming tumor tissues in a “one-size-fits-all” protocol, we established tumor diagnosis-specific dissociation SOPs for optimal 3D tumor spheroid formation in 6-well preculture and 384-well U-bottom plates as described in the Methods section. The detailed protocols for processing vital tumor material are listed in Supplementary Note 2. These protocols mainly differ in the enzymes used and the incubation time at 37 °C. In principle, tissue dissociation consisted of the following steps: (1) extensive mechanical dissociation prior to (2) enzymatic digestion with papain (brain tumors and brain metastases), trypsin (neuroblastomas) or a mix of trypsin and collagenase II for osteosarcomas, soft tissue sarcomas and rare (non-brain) tumor entities; (3) stopping of the enzymatic reaction, digestion of DNA released from dying cells; (4) filtering of the cell suspension, followed by (5) (repeated) red blood cell lysis; and (6) determination of cell number and cell viability (Fig. 3a). We opted for preculturing the cells following tissue dissociation and prior to drug screening for two main reasons, (i) to avoid priming the cells for cell death due to shipment or dissociation-induced stress potentially resulting in false positive drug hits, and (ii) to increase the number of viable cells to allow drug screening, as in many cases the viable cell yield immediately after dissociation was not sufficient to perform a partial (at least one drug plate) or full drug screen (three plates). Depending on the amount of initial material and the speed of recovery or cell expansion, the preculture time varied, with a current median time of four days (mean 6.9 +/− 9.5 days) and with few (seven in total) samples requiring a preculture time exceeding 14 days (Fig. 3b). An outlier sample requiring 77 days of preculturing was not reported to the tumor board. The different diagnoses were distributed across all culture durations, indicating no correlation between the duration of preculture and tumor diagnosis (color code Fig. 3b). Successful pre- and long-term cultures typically resulted in 3D growing spheres with diverse morphology, as shown in Fig. 3c–e. At the drug screening start time, the precultures were dissociated into single-cell solutions (see “drug screening”, Supplementary Note 5) and, after cell counting, transferred onto 384-well preprinted round bottom drug plates to allow for heterogeneous 3D mini tumor spheroid formation (Fig. 3d; Supplementary Figs. 3–9). We aimed to seed 1000 cells per well to achieve comparable conditions. The cell number per well varied between 400/well and 1250/well, depending on the number of viable cells obtained. In 59% of the screens, the intended number of 1000 cells/well could be used (Fig. 3f).

For 66/68 QC-passed samples, the tumor cell content was estimated from the histopathology department for the corresponding fresh frozen tumor. The mean tumor cell content was 78%, and for 48/66 (73%), the tumor cell content was 80% or higher (Fig. 3g). As the tumor cell percentage was lower than 80% in *n* = 18 samples, we analyzed for a subset of samples the composition of the cell population at the time of sample dissociation (passage 0) and time of drug screening (passage 1) with deconvolution assays, such as 850k methylation arrays (Supplementary Data Table 2) and RNA-seq (Fig. 3h, Supplementary Fig. 10a, b). Immune cell type deconvolution and immune/stromal score determination based on RNA-seq revealed a multicellular composition of the original tumor (FF, fresh frozen), the dissociated samples (passage 0) and of the FTC at the seeding time point for the drug screen (passage 1) with a trend for tumor cell enrichment (Fig. 3h, Supplementary Fig. 10a, b). Overall, the fraction of tumor cells remained largely stable or was further enriched during preculture, with ~50–90% tumor cells after dissociation and ~77–87% tumor cell cells at the time of drug screening (Supplementary Data Table 2).

**Fig. 4:**
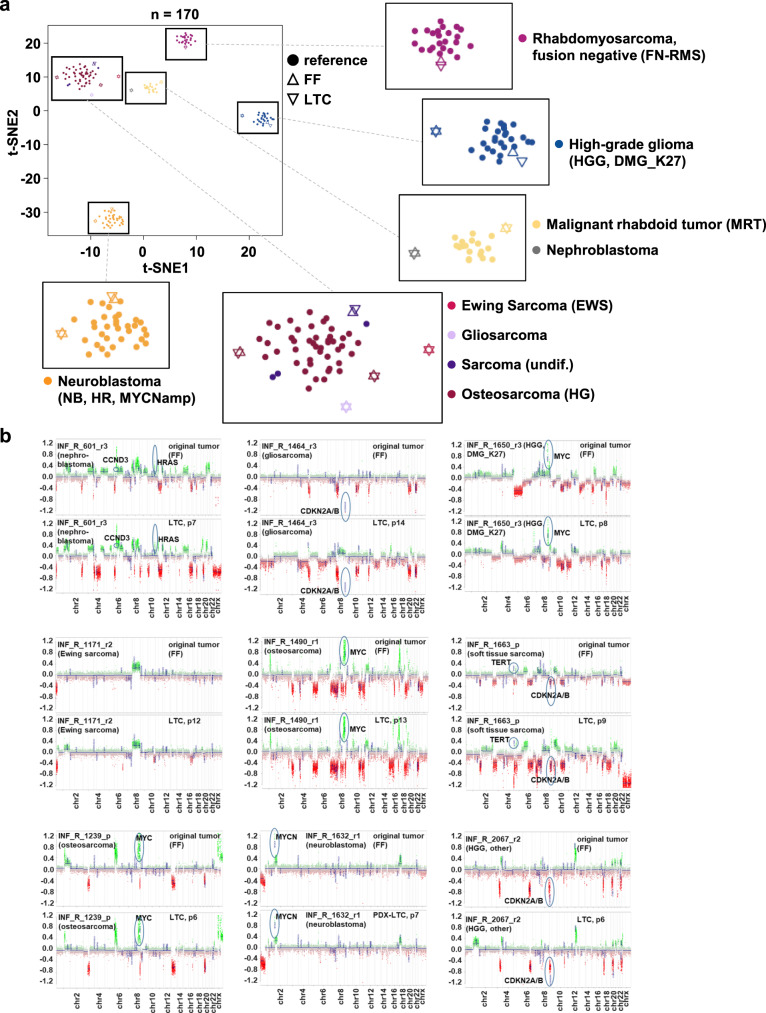
Molecular characterization of the patient-derived 3D culture models. **a** t-SNE analysis of DNA methylation profiles for comparison of the original tumors and the corresponding tumor-derived long-term culture (LTC) models with already existing well-characterized reference tumors (malignant rhabdoid tumors, FN-RMS tumors and high-risk (HR) *MYCN*amp neuroblastomas). **b** Pairwise comparison of the copy-number profiles of nine tumors (upper panel) and their corresponding LTCs (lower panel) reveal similar genome-wide methylation patterns and maintenance of relevant driver events. FF fresh frozen material of the original tumor, LTC long-term culture, EWS Ewing sarcoma, FN-RMS fusion-negative rhabdomyosarcoma, HGG, DMG_K27M high-grade glioma, subtype K27M mutant diffuse midline glioma HGG, other another subtype of high-grade glioma, MRT malignant rhabdoid tumor, NB, HR, MYCNamp high-risk neuroblastoma with *MYCN* amplification, osteosarcoma (HG) high-grade osteosarcoma, sarcoma undiff undifferentiated sarcoma. Sample abbreviations: r relapse, p progression.

The metabolic activity readout was performed after 72 h of drug exposure. To test for genomic stability and identity of the spheroid culture, we have exemplarily compared the copy number profile based on WES and lcWGS data for two samples of FTCs (p. 0/p. 1) with the original tumor (FF) (Supplementary Fig. 11), and the 850k DNA methylation profiles and CNA patterns of passaged long-term cultures (LTCs) with the original tumor (FF) in twelve cases. For the two FTC samples, the RNA-seq analysis confirmed that gene fusions present in the original tumor were also present in the FTCs (Supplementary Figs. 12, 13). Methylation profiling-based t-distributed stochastic neighbor embedding (t-SNE) analysis clustered all LTCs closely with the original tumors. Moreover, all FF/LTC pairs showed high similarities to the corresponding reference methylation classes (diagnoses), as evidenced by grouping with these classes in the t-SNE (Fig. 4a). The maintenance of relevant molecular alterations was confirmed by comparison of the CNA plots for the fresh frozen material from the original tumor (FF) and the corresponding LTC, calculated from the same DNA methylation array dataset (Fig. 4b). Overall, all analyses reveal high concordance between the original tumor sample and corresponding FTC or LTC, indicating the ability of our approach to establish cell culture models closely reflecting the respective patient tumor. This result is especially evident in the maintenance of relevant molecular driver aberrations. All assays reveal an enrichment of tumor cells over passaging time, and the enrichment of tumor cells in the LTC can explain most of the CNA-plot differences. One sample, HGG_DMG_K27, showed a slight separation in the t-SNE but still clustered closely to the reference cohort. In this case, we cannot entirely exclude that we may have established a cell culture reflecting an expansion of a subclone of the original tumor.

Moreover, we used Bland-Altman plots to compare the drug sensitivities of FTCs and LTCs obtained from the same original tumor for two exemplary cases. The models showed similar responsiveness to individual drugs, with only a few outliers above or below the 95% limits of agreement (LoA). The LoA is the average difference ± 1.96 standard deviations of the difference and, hence, is a judgment of how well the measurements agree. The smaller the range is, the better the agreement accuracy will be (Supplementary Fig. 10). Hence, our analyses show that the LTCs closely reflect the original tumor specimens by preserving the molecular diagnoses, genetic driver events, and drug sensitivity patterns.

Overall, we have set up an efficient workflow for handling fresh pediatric solid and brain tumor specimens and established a robust tumor spheroid culture system suitable for drug response profiling.

### Drug sensitivity profiling and tumor board presentation (c)

**Fig. 5:**
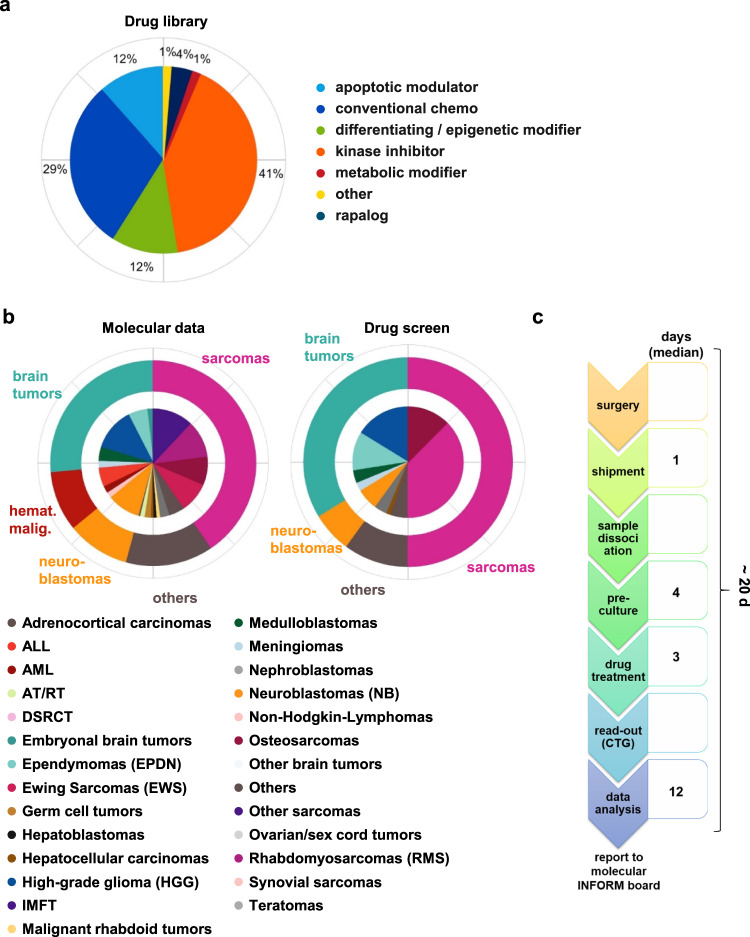
DSP pipeline and cohort overview. **a** Composition of the core drug library, consisting of 75–78 drugs. **b** Cohort overview. Left: Tumor diagnoses of fresh frozen material used for molecular analysis through NGS (*n* = 1642). Right: Tumor diagnosis distribution in the present cohort of INFORM samples with vital tissue submission. The pie diagrams represent the distribution of the indicated diagnoses within the whole cohort. The outer circles represent broad tumor categories: sarcomas (magenta), brain tumors (green), hematological malignancies (hemat. malig., red), neuroblastomas (orange) and others (brown). The inner circle represents the more detailed tumor diagnoses within each category, as explained in the color code below the pie charts. **c** Timeline from surgery to drug report for QC-passed full screens and where timeline data were available (*n* = 49). ALL acute lymphocytic leukemia, AML acute myeloid leukemia, AT/RT atypical teratoid rhabdoid tumor, DSRCT desmoplastic small-round-cell tumor, EPDN ependymoma, EWS Ewing sarcoma, HGG high-grade glioma, IMFT inflammatory myofibroblastic tumor, NB neuroblastoma, RMS rhabdomyosarcoma, QC quality control.

Due to the limited availability of tissue under real-world clinical conditions, we used a clinically focused drug library (*n* = 75–78 drugs) (Supplementary Data Table 3), established within the COMPASS (Clinical implementation Of Multidimensional PhenotypicAl drug SenSitivities in pediatric precision oncology) cooperation covering most of the standard chemotherapeutic drugs used in pediatric oncology treatment protocols and representative small molecular kinase inhibitors, epigenetic modifiers, apoptotic modulators, metabolic modifiers and others (Fig. 5a). Most of the drugs are either European Medicines Agency (EMA) or U.S. Food and Drug Administration (FDA) approved or in late-stage clinical development. All drugs were administered batchwise on 384-well U-bottom plates and stored in an oxygen- and moisture-free environment at room temperature to allow on-demand availability before the seeding of cells.

During the first 24 months of the INFORM DSP pilot program, in 89/132 (67%) of cases, a sufficient volume of viable tissue was received, allowing a partial or full library screen to be performed. During this period, INFORM received 998 fresh frozen tissue samples for NGS. Thus, 13% (132/998) of all INFORM cases submitted for genomic profiling had accompanying viable tissue submitted for drug sensitivity profiling (DSP). The pie diagrams in Fig. 5b display the distribution of broad categories (outer circle) and more detailed tumor diagnoses within these categories (inner circle) for DSP. The most frequent categories were sarcomas (44% soft tissue sarcomas and Ewing sarcomas and 11% osteosarcomas), followed by brain tumors (29%; high-grade gliomas, ependymomas, medulloblastomas and others), neuroblastomas (9%) and a mixed group of rare tumor entities (7%). This distribution of viable tissue samples for DSP reflects the overall distribution of categories and tumor diagnosis in the total INFORM cohort (*n* = 1642 at the time of data cutoff for this study). The submission of fresh viable tumor specimens is optional when including patients in INFORM, whereas sending fresh frozen material for NGS is mandatory. Thus, some samples, in particular rare tumor diagnoses (i.e., inflammatory myofibroblastic tumors, synovial sarcomas or atypical teratoid rhabdoid tumors) or samples with generally smaller specimen sizes due to difficult surgical approaches, may be underrepresented in the DSP cohort. DSP results from the QC passed drug screens were reported together with the respective NGS data at the weekly INFORM molecular tumor board meeting. The timeline in Fig. 5c illustrates the individual steps of the DSP workflow and corresponding median durations during the pilot phase, with drug treatment fixed at 72 h. The overall median turnaround time of the DSP from surgery to data analysis was 20 days (mean: ~24 days), with a median data analysis time of 12 days (mean: 12.7 +/− 8.5 days). The ‘data analysis’ time also includes the interpretation of the data.

Drug hits were identified after accounting for the following: (i) The drug sensitivity score (DSS_asym_)^13,14^, adjusted for effects on a set of healthy bone marrow control samples as published in Pemovska et al.^9,15^ plus in-house controls (nonmalignant astrocytes and fibroblasts); (ii) the maximal effect of the drug, which should reach 75% inhibition or more; (iii) the absolute IC50, which should be lower than the in vivo C_max_ concentration; and (iv) the goodness of fit (*R*^2^) for the calculated growth curve, which should be 0.8 or higher. In addition, the cohort dot plot detected above-average DSS for individual samples, and the 75th percentile was considered to indicate an above-average response within the cohort. We only reported drugs already approved or in clinical studies to the tumor board. The effects of investigational drugs were considered as confirmation of in-class drug effects, if applicable.

**Fig. 6:**
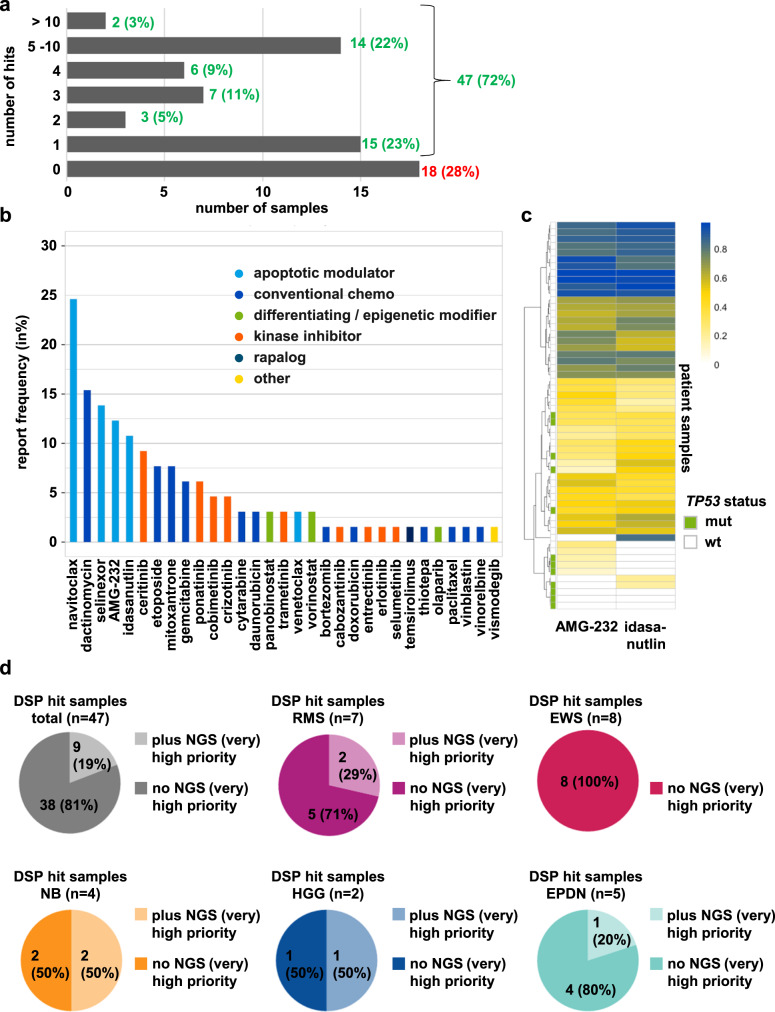
Identified drug hits reported to the INFORM molecular tumor board. **a** Bar diagram displaying the number of reported hits per sample and in %. **b** Bar diagram depicting the report frequency (in %) of single drugs and drug classes. Color code integrated. **c** Unsupervised hierarchical clustering based on DSS_asym_ quantiles for the MDM2 inhibitors AMG-232 and idasanutlin. The color scale shown at the top represents quantile values. The *TP53* status is highlighted with green (mutant) and white (wild-type) bars on the left side. **d** Pie diagrams reflecting the proportion of samples with reported hits with or without reported NGS (very) high-evidence targets for the total DSP cohort and different diagnoses.

Among 65 (without repetition) successfully screened INFORM samples, 47 (72%) demonstrated at least one drug hit, with some samples exhibiting even 10 or more hits. We could not identify any drug hits for 18 samples (28%) (Fig. 6a). The most frequently reported drug class was apoptotic modulators (e.g., navitoclax), followed by conventional chemotherapy (Fig. 6b). Samples demonstrating sensitivity to the pan BCL2 family inhibitor navitoclax were, in general, also sensitive to the BCL2 selective inhibitor venetoclax and other investigational selective BCL2 family inhibitors (Supplementary Fig. 14a; Supplementary Data Table 4). However, in most cases, venetoclax did not qualify as a hit due to a percent inhibition at C_max_ below the 50% cutoff, except for two of four neuroblastoma samples. This finding is in line with cell culture and preclinical data from several research groups, which demonstrated the strong effectiveness of BCL2 inhibitors in neuroblastoma models. Hence, venetoclax is currently being evaluated in clinical phase I trials for treating neuroblastoma (clinicaltrials.gov NCT03236857)^16–19^.

To analyze whether the response to BCL2 inhibitors is linked to the expression of distinct BCL2 family members, we performed a Manova analysis of samples with the highest response (DSS_asym_ quantile >75%) versus less responsive samples. The analysis identified proapoptotic *BBC3 (PUMA)* and *BCL2L11 (BIM)* as significantly upregulated in responsive samples when comparing BCL2 inhibitor-responsive versus less-responsive samples (Supplementary Fig. 14b, c), indicating the expression of these genes as potential biomarkers for response prediction. Conversely, high expression of the antiapoptotic *BCL2L12* significantly correlated with a low response to these drugs (Supplementary Fig. 14b, c).

**Fig. 7:**
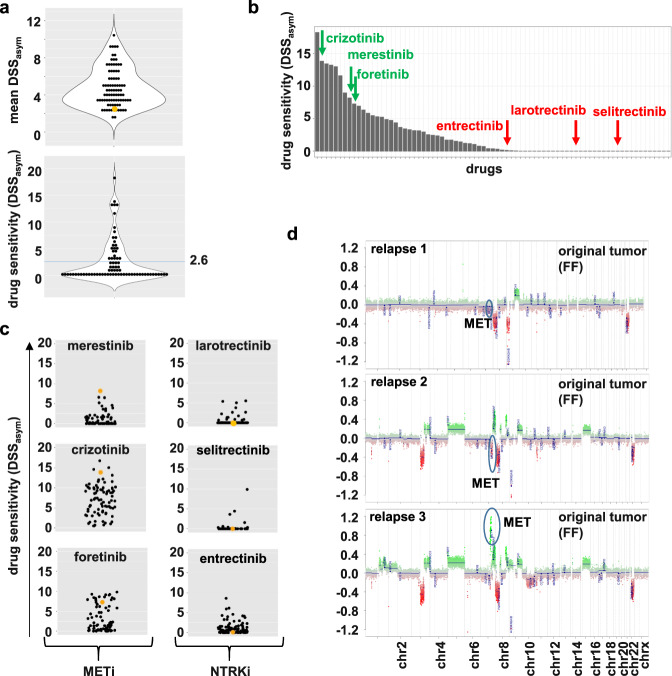
Case report #1: *NTRK* fusion-positive high-grade glioma with acquired resistance to NTRKinhibitors due to *MET* amplification. **a** Violin dot plots displaying the mean DSS_asym_ values for the whole cohort (upper graph) and each drug for the respective sample (lower graph; blue line: mean). The sample of interest (here, case #1 relapse 3) is marked in orange. **b** Waterfall plot sorting all tested drugs for the case #1 relapse 3 sample upon their DSS_asym_ values, starting with the highest on the left. **c** Dot plots depicting the DSS_asym_ values for the indicated drugs for all successfully screened samples. The sample of interest (here, case #1 relapse 3) is marked in orange. All three MET inhibitors (merestinib, crizotinib, foretinib) show above-average responses, whereas the three NTRK inhibitors all have a DSS_asym_ of or close to 0. **d** Copy-number profiles of the FF samples reveal an acquired (or selected) *MET* amp in the plot for relapse 3.

As a first step toward evaluating our DSP platform’s clinical utility and predictivity, we looked at cases with strong genetic driver alterations identified by molecular profiling and the vulnerability to matching drugs. Indeed, in 9/14 patient cases, we identified at least one drug sensitivity hit matching the tumor-driving alteration. Of the five cases with no hit identified, one had an NTRK-fusion with clinical resistance to several lines of TRK-inhibitors (see also Fig. 7), and three harbored a CDK4/6 amplification known to confer resistance to CDK4/6 inhibitors^20^ (summarized in Supplementary Data Table 5). The matching drug sensitivity becomes especially evident when comparing the sensitivity of the sample of interest to the response of the rest of the DSP cohort, visualized in the DSS_asym_ quantile waterfall plots (Supplementary Fig. 15; molecular aberration matched drugs are marked with arrows). NGS matching drugs present with the highest quantile ranks. Furthermore, DSP gives additional value to NGS target base identification of vulnerabilities, as it gives information on which drug of the respective drug class is more effective (Supplementary Fig. 15).

To further validate our platform, we made use of the fact that *TP53*-wild-type tumors can be responsive to MDM2 inhibitors, as the tumor suppressor function of p53 is frequently impaired due to enhanced activity of its upstream negative regulator MDM2. However, tumors with inactivating mutations in *TP53* are resistant to most MDM2 inhibitors (reviewed in refs. ^21–23^). To determine whether this correlation was reflected in our DSP results, we visualized MDM2 inhibitor (idasanutlin and AMG-232) sensitivity (determined as DSS_asym_ quantiles) and the *TP53* status of our DSP samples in an unsupervised hierarchical clustering heatmap. Samples harboring a *TP53* mutation were less sensitive to MDM2 inhibitors than *TP53* wild-type samples. Moreover, samples with clear above-average idasanutlin and AMG-232 sensitivity ranking in the top 25% of the cohort (above 75% quantile, blueish color; Fig. 6c) were exclusively *TP53*-wildtype tumors. Conversely, samples exhibiting resistance to MDM2 inhibition within the cohort predominantly harbored *TP53* mutations. Quantile waterfall plots for representative cases with different diagnoses are depicted in Supplementary Fig. 16.

Importantly, in ~81% (38/47) of all successfully screened INFORM cases with a reported drug hit (*n* = 47), a drug hit was present in samples in which WES and RNA-Seq did not identify an actionable target with a high or very high evidence level^1^ demonstrating added information from ex vivo DSP in the majority of cases. This finding especially holds true for tumor diagnoses in which no or only a few very high or high evidence level targets^1^ are typically detected, such as ependymomas, rhabdomyosarcomas or, most evident, Ewing sarcomas (Fig. 6d). An exemplary DSP result, visualized as a DSS_asym_ quantile plot, for a nephroblastoma sample for which only borderline or very low evidence targets^1^ (*BCL2*, *XPO,* and *HDAC2* overexpression) were identified is shown in Supplementary Fig. 15. In addition to detecting drugs matching the identified molecular alterations (marked by arrows), namely, BCL2 inhibitors navitoclax and venetoclax), XPO inhibitor selinexor and all four HDAC inhibitors present in the library), the DSP demonstrates an unexpected in-class effect for all MEK inhibitors in the library, ranking with the highest quantiles for this sample (marked with asterisks). Biocomputational analyses addressing the underlying (molecular) mechanism of this unexpected sensitivity to MEKi are ongoing.

Overall, our INFORM drug sensitivity pipeline can identify drug hits, matching corresponding molecular driver alterations and, more importantly, can identify unexpected drug sensitivities in a high proportion of pediatric solid and brain tumors lacking clinically relevant molecular targets.

### Selected clinical case reports demonstrating ex vivo–in vivo correlation of drug sensitivity profiles

To look into potential correlations between the output of our ex vivo drug sensitivity platform and clinical courses of patients in vivo, we evaluated samples from three patients for whom clinical follow-up data were available. Figure 7 demonstrates case #1, a seven-year-old patient with high-grade glioma enrolled in INFORM in whom a *BCR:NTRK2* fusion was identified by NGS (relapse 1). Consequently, the patient was enrolled in an NTRK-inhibitor trial (larotrectinib) and, following progression (relapse 2), a 2nd generation NTRK inhibitor trial (selitrectinib). Following further progression (relapse 3), the patient received another biopsy to obtain tissue for molecular analysis and ex vivo DSP, which passed the QC. Overall, the tumor sample was quite resistant, with a mean DSS_asym_ below 3.0 (Fig. 7a), and showed complete resistance against all NTRK inhibitors in the library (larotrectinib, selictretinib, and entrectinib), reflecting the clinical course of the patient. However, the sample exhibited high sensitivity to several MET-targeting inhibitors of the library, namely, merestinib, crizotinib, and foretinib (Fig. 7b, c; Table 1). Subsequent NGS analyses revealed an acquired (or selected) *MET* amplification as a likely resistance mechanism to NTRK inhibition, which is consistent with the drug screening results (Fig. 7d). This finding is in line with other recent findings describing acquired *MET* amplification as a potential resistance mechanism to NTRK inhibitor therapy, similar to that described for targeted EGFR inhibitor therapy in NSCLC patients^24,25^. In addition, unexpected drug hits were identified in this case, including sensitivity to ALK (lorlatinib) and JAK1/2 (ruxolitinb) inhibitors.

**Table 1:** Drug hits with above-average drug response (quantile ≥ 75%) for case #1 relapse 3 (HGG).

Ranking	Drug name	Drug target	DSS_asym_ quantiles^a^
1	merestinib	METi	98.4%
2	crizotinib	METi, ALKi	93.7%
3	ruxolitinib	JAK1/2i	87.3%
4	cabozantinib^b^	METi	77.2%
5	lorlatinib	ALKi	77.2%
6	foretinib	METi	75.4%
7	thioguanine	Conv. chemotherapy (DNA)	75.4%

^a^A quantile for DSS_asym_ ≥ 75% points toward an above-average response within the cohort. DSS_asym_; drug sensitivity score.

^b^DSS_asym_ < 5.

**Fig. 8:**
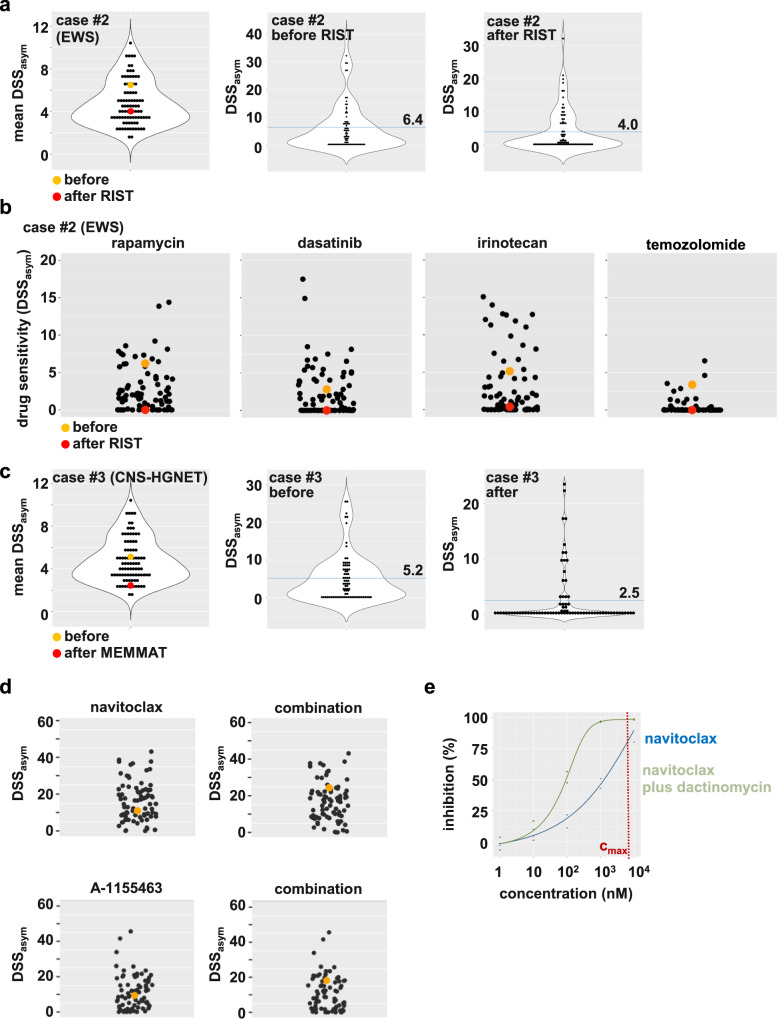
Case reports #2 and #3: Evolution of treatment-associated drug resistance in serial sample collections. **a** Violin dot plots of case #2, a relapsed *EWSR1:FLI1*-positive Ewing sarcoma (EWS) before and after RIST therapy. The dot plots display the mean DSS_asym_ values for the whole cohort (left) and each drug for the respective sample before RIST treatment (middle) and after RIST treatment (right). The sample of interest before RIST is marked in orange, and the sample after RIST is marked in red. The blue line represents the mean. **b** Drug dot plots depicting the DSS_asym_ values for the indicated drugs for all successfully screened samples. The sample of interest before RIST is marked in orange, and the sample after RIST is marked in red. All four drugs displayed a strong decrease in DSS_asym_ after RIST therapy. **c** Violin dot plots of case #3, a relapsed CNS-HGNET brain tumor. Shown are DSP 1 and DSP 2 before and after MEMMAT therapy. The dot plots display the mean DSS_asym_ values for the whole cohort (left) and each drug for the respective case (DSP 1 in the middle; DSP 2 on the right). The sample of interest DSP1 before MEMMAT is marked in orange, and DSP2 after MEMMAT is marked in red. The blue line represents the mean. **d** Drug dot plots depicting the DSS_asym_ values for the single treatment (navitoclax or BCL-XL inhibitor A-1155463) and the combination (navitoclax plus dactinomycin or A-1155463 plus dactinomycin). The sample of interest (case #3, CNS-HGNET) is marked in orange. **e** Dose-response curves of a single compound (navitoclax) and combinatorial treatment (navitoclax plus IC_25_ 10 nM dactinomycin). The overlay of both curves, reflecting a shift in sensitivity upon combinatorial treatment, is depicted. The combination screen was performed with cryopreserved cells cultured for four days after thawing for drug testing. % inhibition: normalized inhibition of metabolic activity.

In case #2 with relapsed *EWSR1:FLI1*-positive Ewing sarcoma, serial viable tissue sampling with DSP before and after therapy allowed us to monitor the evolution of drug resistance under multiagent chemo- and targeted therapy. The first sample for DSP was obtained after the 11-year-old patient experienced relapse during treatment according to the EWING2008 study protocol (before RIST). The patient then received a RIST multidrug treatment regimen (rapamycin, irinotecan, dasatinib (Sprycel), temozolomide)^26^, and was biopsied again fourteen months later with a further relapse (after RIST). Figure 8 shows a shift of drug sensitivity from a more sensitive profile (mean DSS_asym_ 6.4) to a generally drug-resistant profile (mean DSS_asym_ 4.0) (Fig. 8a). In particular, DSP revealed the emergence of complete resistance to all four RIST regimen drugs at the second relapse, collected after RIST therapy (Fig. 8b; Table 2). In this case, we identified HDAC inhibitors (e.g., entinostat) as drug hits with potential clinical use and the investigational BET inhibitor I-BET151.

**Table 2:** Drug response details for cases #2 (EWS) and #3 (CNS-HGNET-MN1).

Case #2	DSS_asym_ quantiles before RIST^a^	DSS_asym_ quantiles after RIST^a^
Temozolomide	91.2%	0%
Rapamycin (Sirolimus)	82.5%	0%
Irinotecan	79.4%	46.0%
Dasatinib (Sprycel)	63.5%	0%

Case #3^b^	DSS_asym_ quantiles relapse 3	DSS_asym_ quantiles relapse 4
Etoposide (used for treatment before 3rd and between 3rd and 4th relapse)	46.0%	0%
Cytarabine (used for treatment between 3rd and 4th relapse)	25.4%	0%
Selinexor (reported drug hit for relapse 3)	80.7%	56.1%
Dactinomycin (reported drug hit for relapse 3)	77.2%	0.1%
Navitoclax (best hit for relapse 4)	44.4%	73.0%

^a^RIST multidrug treatment regimen: rapamycin, irinotecan, dasatinib (Sprycel), temozolomide.

^b^Fresh tumor tissue was received from relapses three and four. Between these two relapses, the patient was treated according to the MEMMAT therapy regimen, including etoposide and cytarabine.

Case #3 relates to a patient diagnosed with a CNS-HGNET-MN1 tumor at the age of nine. In the following years, two relapses were surgically removed, the patient received proton therapy and, after the second relapse, a six-month chemotherapy regimen with etoposide and trophophosphamide. We received fresh tumor tissue from relapses three and four (at 13 and 14 years of age, respectively). Between these two relapses, the patient was treated according to the MEMMAT (Medulloblastoma European Multitarget Metronomic Anti-Angiogenic Trial)^27^ therapy regimen, which included etoposide, cyclophosphamide, and cytarabine (Table 2). The two DSPs revealed a similar shift in the mean DSS_asym_ as case #2 from 5.2 to 2.5 (Fig. 8c), indicating the emergence of multidrug resistance after treatment. As dactinomycin was one of the top hits reported for the first sample (Table 2), we performed a combination screen with dactinomycin (IC_25_: 10 nM) against our standard drug library. Potential beneficial combination partners for dactinomycin were identified with the differential combination drug sensitivity score dcDSS_asym_^13^, which corresponds to the largest difference between the DSS_asym_ values for each drug in the presence and the absence of 10 nM dactinomycin (Supplementary Data Table 6). The BCL2 family inhibitor navitoclax showed the largest shift in drug sensitivity (dcDSS_asym_ 13.4), supported by the in-class effect of the investigational BCL-XL inhibitor A-1155463 (dcDSS_asym_ 9.3; Fig. 8d). Venetoclax, a selective BCL2 inhibitor, failed to substantially increase drug sensitivity (dcDSS_asym_ 0.8), indicating a functional role of BCL-XL and not BCL2 in the observed combination activity. This result is also evident from the overlay of the drug response curves for navitoclax in the absence and presence of dactinomycin and the almost complete inhibition of metabolic activity at C_max_ of 98% through the combination (Fig. 8e).

Overall, these cases demonstrate striking parallels between clinical courses and our ex vivo functional precision medicine platform, suggesting an “imprinting” of drug resistance patterns in the ex vivo drug sensitivity profiles; however, further prospective evaluation of the predictivity of our DSP platform in a larger patient cohort is needed.

## Discussion

To the best of our knowledge, this is the largest pediatric clinical series on functional precision oncology covering a broad spectrum of solid and brain tumor entities, including the implementation of results in a molecular precision oncology tumor board.

Due to the real-world, multinational, multicenter setting of this study, we could investigate the impact of several critical prescreening parameters on the screening success rate, including the mode of tissue collection condition, tissue sample size and tissue shipment conditions. Based on our data, we propose a simple protocol, summarized in Supplementary Fig. 17. A tumor tissue piece ideally larger than 250 mm^3^ (~250 µl) should be shipped in serum-free culture medium at room temperature within 48 h post-surgery. If culture medium is not available in the operation room, physiological saline solution can be used as an alternative. Notably, smaller tissue pieces, e.g., from fine needle biopsies, do not exclude screening success per se. This protocol enables (i) small centers without specific laboratory facilities to participate in functional precision oncology programs, which is particularly important for studies with small patient populations, such as pediatric oncology, and (ii) drug sensitivity profiling (DPS) of limited tissue material from small biopsies, including stereotactic biopsies. We could successfully process and screen over 50% of all submitted viable tissue samples and present the results at the INFORM weekly molecular tumor board meeting, although this study included only brain and solid tumors, which are considered rather difficult to process in such a program^7^.

Patient-derived 3D fresh tissue culture preserves tumor heterogeneity and response to drugs^10^. We have exemplarily confirmed the genomic characteristics of tumor cells using WES and lcWGS data in two FTC samples at two time points (after dissociation and at drug screening) and 850k DNA profiling in twelve samples cultured over at least six passages, ensuring that within the time frame of preculture and drug screening, tumor cells keep their genomic profile reflecting the original tumor. Even more importantly, the composition of tumor heterogeneity was preserved (with a trend for tumor cell enrichment) during our preculture and screening conditions with no evidence of overgrowth of, e.g., stromal cells during the course of the drug screening protocol, as demonstrated by RNA-seq and deconvolution approaches. Thus, our preculture protocol allows us to screen the tumor cell sample within a multicellular composition, resembling the tumor microenvironmental condition more closely than completely purified cancer cell lines, which loose most aspects of tumor heterogeneity and lack microenvironmental signals potentially affecting drug response.

Furthermore, the generation of long-term cultures (LTCs) that reflect the original tumor specimen by preserving the genetic driver events and DSP provides a unique source for analyses, especially for novel models derived from rare tumor entities or tumors with very specific molecular alterations (e.g., fusions). These molecularly defined models will allow detailed functional studies, such as those identifying mechanisms underlying therapy sensitivity (biomarker) or resistance.

Recently, the first results of the TARGET pilot study of the Australian ZERO Precision Childhood Cancer Program have been published by Lau and colleagues^28^. Similar to our study, pediatric patients with high-risk poor prognosis cancer were enrolled, covering a broad spectrum of pediatric solid and brain tumors. The study included two centers from Sydney as opposed to our multinational, multicenter study including 35 sites from seven countries. Our INFORM study included 132 samples undergoing drug screen attempts versus 46 samples in the TARGET/ZERO study. One of the largest differences between Lau et al. and our study is the median “expansion time” of tumor material (ZERO, 4.3 months versus INFORM, 7 days) required for drug screening, reflecting the fact that the majority of tumor cells underwent expansion procedures (either in vitro or through PDX mice) in the TARGET/ZERO study before drug screening. Consistently, direct drug screening of tumor samples within the first 14 days could be performed for 5/46 (11%) in TARGET/ZERO due to a lack of sufficient cell numbers as opposed to 82/132 (62%) in our series. This time difference is significant for clinical decision-making, as the population with relapsed high-risk pediatric cancers has a median progression-free survival time of only four months^1^. The sixfold higher success rate for upfront drug screening in our study can be mainly explained by the available tissue sample sizes, likely reflecting different surgical approaches in the collaborating centers. Drug hits were identified in 13/17 (76%) cases in the TARGET/ZERO study, which was very similar to 47/65 (72%) in our INFORM series despite both programs using different drug-hit calling algorithms. Notably, in both studies, an added value of DSP was demonstrated in a high proportion of tumors with no detectable targets following molecular profiling. Conversely, in tumors with detected actionable targets, molecularly matching drug hits could be detected in a significant proportion by DSP, including tumors with BRAF, ALK, MET, NTRK driver alterations, BCL2 family expression and TP53 status. The match of drug hits and targets is especially evident when comparing the DSP of the sample of interest to the whole cohort. Hence, during our pilot phase, we successively included outlier responses with respect to the DSP cohort (above-average response) in our hit selection process. This approach is in line with that of the ZERO/TARGET study, which also used a cohort-based approach for hit identification. Although promising correlations of ex vivo DSP with clinical courses were reported in both studies, these data are limited at this point due to the low sample size and correlative nature of the analysis warranting further prospective testing in interventional trials.

Nevertheless, we have already made some striking clinical case observations. A patient with NTRK fusion-positive high-grade glioma progressing after first- and second-line NTRK inhibitor therapy revealed complete resistance of tumor tissue to all NTRK inhibitors in ex vivo DSP. Moreover, MET inhibitors were identified independently of NGS results as top-ranked sensitive drugs. In parallel, acquired *MET* amplification as a resistance mechanism to NTRK inhibitor therapy was detected by NGS. In a second and third case, we could compare DSPs of the same tumor before and after multiagent therapy, demonstrating the emergence of ex vivo drug resistance paralleling the patient’s clinical course. These cases demonstrate the importance of obtaining tumor samples from the current disease episode for functional precision oncology platforms since any therapeutic intervention appears to significantly impact drug sensitivity and resistance patterns; thus, clinical decisions should not be made on “historical” or archived viable tumor samples.

Our data clearly demonstrate the additional benefit of ex vivo DSP in the era of precision oncology. In 80% of the cases in our pilot study, DSP identified drug hits in tumors that lacked actionable high-evidence-level drug targets according to our INFORM seven-scale prioritization scheme we have published recently^1^. Moreover, Gatzweiler et al.^29^ showed that the pipeline can be extended for rapid in vivo drug screening with zebrafish-PDX models from primary material obtained through the INFORM pediatric precision oncology pipeline.

A recent case report demonstrated the clinical benefit of DSP in a child with refractory rhabdomyosarcoma receiving DSP-informed chemotherapy^30^. More systematically, DSP has been described in several adult precision oncology studies^7–9,31^. Despite differences in tissue processing, culture conditions (2D versus 3D), drug screening formats (bulk ATP measurements versus single-cell imaging) and bioinformatic processing (DSS versus AUC z scores) of data, similar to our conclusion, drug hits have been identified in a substantial proportion of samples lacking actionable alterations.

In contrast to other reports, which are based on single institutional patient enrollment, our DSP platform was established in an international, multicenter setting, which is important to achieve a critical number of samples in rare diseases such as pediatric cancers. We are currently testing the reproducibility of DSP results using metabolic and imaging readouts across different European platforms in a consortium involving our institution and institutions from France, the Netherlands, Finland and Hungary, termed COMPASS (https://www.kitz-heidelberg.de/en/the-kitz/kitz-newsroom/kitz-news/detail/compass-the-guide-to-new-therapies-for-children-with-cancer/), aiming for the harmonization of DSP pipelines.

In contrast to organoid-based or PDX-based reports^7,8^, our pipeline was optimized to allow real-time acquisition of DSPs and reporting of results to the molecular tumor board with a quick turnaround of ~3 weeks from tissue arrival to the final data report. This fast process enabled us to promptly discuss the cases with NGS target data with the molecular tumor board. Organoid-based culture models require relatively long culture periods (>5 passages) before drug screening can be performed^7^. This long culture period delays the availability of DSP data for clinical decision-making in comparison to our platform, which can be disadvantageous, as most tumors grow aggressively with a mean patient PFS duration of only four months in the INFORM registry population^1^.

Recently, two studies have demonstrated the clinical benefit of functional precision oncology in adult patients with hematological malignancies^9,31^. Although both studies have used different platforms (single-cell high-content microscopy and bulk metabolic assays, the authors showed significantly higher patient survival rates compared with previous treatment of the same patient as an internal control^31^ and high response rates, including complete responses^9^.

In summary, our INFORM functional precision oncology pilot study for children with relapsed solid tumors demonstrates that ex vivo DSP in a multicenter, real-world setting is feasible with a short turnaround time to the molecular tumor board. We provide evidence that DSP may provide additional value to NGS-based molecular diagnostics, particularly in pediatric brain tumors and sarcomas lacking actionable molecular alterations. Prospective evaluation of the predictivity of ex vivo DSP with clinical outcome in an interventional clinical trial is the next logical step in the investigation of the true clinical potential of functional precision platforms in pediatric oncology.

## Methods

### Primary cell isolation from fresh tumor tissue

Incoming tissue piece sizes varied significantly depending on the extent of surgical resection performed as part of standard patient care. The samples were measured in three dimensions with a ruler directly in the INFORM sample receiving laboratory.

Upon receipt of the fresh surgical specimen, the shipment solution (saline or cell culture medium) was discarded, if applicable, after spinning down small tumor fragments, and all tumor pieces were transferred to a sterile 10 cm cell culture dish with as little liquid as possible. Macroscopically visible blood coagulates were cut off, and the tumor tissue was chopped with sterile scalpels or scissors to obtain small pieces. The mechanically homogenized tissue was subjected to subsequent enzymatic digestion and further processing according to protocols adapted from Eisemann et al.^32^ for brain tumors and brain metastases and Stewart et al.^33^, Kodack et al.^34^, and Pauli et al.^8^ for all other tumor diagnoses (for details, see “processing of vital tumor material”, Supplementary Note 2). Briefly, enzymatic digestion was performed with papain (brain tumors and brain metastases), trypsin (neuroblastomas) or a mix of trypsin and collagenase II (all other tumor entities) and stopped after 10–90 min, depending on the tumor diagnosis. DNA released from dying cells was removed by adding DNAse I, and the resulting cell suspension was filtered to obtain single cells and small cell aggregates. Red blood cells were lysed if their presence was clearly visible, except in cases where the tumor specimen/the resulting cell pellet was very small, and centrifugation steps were reduced to save material. The addition of fetal bovine (or other) serum was omitted throughout the sample preparation. The cells were resuspended in TSM complete^35^, counted and cultured at a density of up to 3–4 × 10^6^ cells per ml of TSM complete (for “medium recipes” see Supplementary Note 4). For cell counting, either ViCell XR (Beckmann Coulter) or, starting from August 2020, Cellometer K2 (Nexcelom Bioscience) was used (for more details on “primary cell culture” see Supplementary Note 3, for more details on cell counting and seeding, see “drug screening and metabolic activity assay”, Supplementary Note 5). The cells were closely monitored by light microscopy in the days following tumor dissociation. Most cultures formed free-floating three-dimensional spheroids within 24 h after tumor dissociation. In some cases, we observed semiadherent spheroids or adherent cell cultures. We chose the time point to seed the cells for drug screening based on the morphology and growth behavior of the cultures. We aimed to subject the cultures to drug screening two to seven days after tumor dissociation.

### Generation of long-term patient-derived cell cultures

Long-term cultures (LTC) in serum-free TSM complete medium were established as described previously^36^. Briefly, when the spheroids reached a diameter of ~700–1000 nm, or the cultures were confluent, the cells were subcultured by dissociation with TrypLE express (12604013; Life Technologies) and seeded at a ratio of 1:2 to 1:5 in fresh TSM complete. The cultures were considered as an established LTC when they surpassed at least ex vivo passage six. The absence of interspecies contamination, mycoplasma, squirrel monkey retrovirus and Epstein-Barr virus was confirmed by multiplex cell contamination test (McCT; Multiplexion, Heidelberg, Germany) as described in Schmitt & Pawlita^37^. Maintenance of the molecular characteristics of the original tumor was validated as described below.

### Molecular diagnosis

The molecular diagnosis, methylation profile, and copy number aberrations for patient-derived samples were assessed with the Infinium MethylationEPIC Bead Chip (Illumina) according to the manufacturer’s instructions. The data were used for molecular classification by comparison with an in-house reference set for sarcomas^38^, an in-house reference set for HGGs (DGM-K27)^39^ and an in-house reference set for high-risk (HR) *MYCN*-amplified neuroblastomas^40^, and compared to the respective data of the original tumor.

WES, lcWGS, and RNA-seq data were generated as described previously^1^. Briefly, DNA and RNA were prepared from fresh frozen tumor (FF) material, from fresh tumor tissue (FTC) after sample dissociation or from spheroid cell cultures at different time points using QIAamp® DNA Mini Kit (Qiagen) and NucleoSpin RNA Kit (Macherey Nagel), respectively, according to the manufacturers´ instructions. DNA and RNA quantity were measured with a Qubit Fluorometer using the respective Qubit assays (Invitrogen). DNA and RNA quality were evaluated via TapeStation Analysis (Agilent Technologies). Paired-end libraries from tumor and germline DNA were prepared using either the Agilent SureSelectXT Human V5 kit or the Agilent SureSelect XT HS + Human All Exon V7 for whole-exome sequencing (WES) and, with the exclusion of the enrichment step, low-coverage whole-genome sequencing (lcWGS; on average 1x genome coverage). Libraries were sequenced on the Illumina HiSeq4000 or NovaSeq 6000 systems, in paired-end mode with typically 101 bp read length. Strand-specific RNA sequencing libraries were prepared using the Illumina TruSeq protocol (Illumina TruSeq Stranded RNA kit) and sequenced on the Illumina HiSeq4000 or NovaSeq 6000 HiSeq system in paired-end mode, with ~80 million reads from paired-end sequencing. All samples were submitted to the Genomics and Proteomics Core Facility (GPCF) of the German Cancer Research Center (DKFZ) for low-coverage WGS, WES and RNA sequencing and were only included for library preparation after passing all standard quality controls.

For immune microenvironment analysis, we used ESTIMATE with default parameters to measure overall immune infiltration (https://bioinformatics.mdanderson.org/public-software/estimate/)^41^. We used the TIMER2^42^ web interface (http://timer.cistrome.org/) for comprehensive analysis of immune-cell composition using six computational tools, CIBERSORT, EPIC, QUANTISEQ, MCPCOUNTER, TIMER, XCELL.

To estimate immune cell composition using ICGC DNA methylation array data, we used the EPIDISH R package^43^ with default parameters using reference methylation signatures as previously reported^44^.

The following reporters were applied for the BCL2 family gene expression analysis (RNAseq, platform gencode19; dataset: Tumor Pediatric Inform - Pilot / Registry - 1642): *BAD*, ENSG00000002330.9; *BAK1*, ENSG00000030110.8; *BBC3*, ENSG00000105327.11; *BCL2*, ENSG00000171791.10; *BCL2A1*(*BFL1*), ENSG00000140379.7; *BCL2L12*, ENSG00000126453.5; *BCL2L13*, ENSG00000099968.13; *BCL2L14*, ENSG00000121380.8; *BCL2L15*, ENSG00000188761.7; *BCL-W* (*BCL2L2*), ENSG00000129473.5; *BCL-XL* (*BCL2L1*), ENSG00000171552.8; *BID*, ENSG00000015475.14; *BIK*, ENSG00000100290.2; *BIM* (*BCL2L11*), ENSG00000153094.17; *BMF*, ENSG00000104081.9; *MCL1*, ENSG00000143384.8.

### Drug screening and metabolic activity assays

Drug screening was performed in round bottom 384-well format to allow for the formation of 3D spheroids. The drugs were administered to the plates before seeding and consisted of 75–78 clinically relevant anticancer drugs (targeted as well as chemotherapy) in five concentrations spanning five orders of magnitude, with each condition tested in duplicate and maximum (benzethonium chloride), intermediate (sublethal concentration of staurosporine (250 nM) and minimum (DMSO) effect control wells (prepared at FIMM High Throughput Biomedicine Unit, FIMM, HiLIFE, University of Helsinki, Finland)^9,15^. Plates were stored in an oxygen- and moisture-free environment at room temperature (San Francisco StoragePod, Roylan Developments, Fetcham Leatherhead, UK) until use.

The quality control classification depends primarily on the computed robust Z’, assessing the separation of controls (positive and negative controls). The robust Z’ is calculated based on the dispersion of the control mean absolute deviation (mad) and the control median, which is less sensitive to outliers.

The normalized inhibition of metabolic activity is depicted as % inhibition and is calculated as follows: the dose responses per drug and concentration are normalized to mean negative (DMSO) and mean positive (benzethonium chloride) controls per plate, resulting in relative inhibition values ((raw count sample−mean DMSO)/(mean benzethonium−mean DMSO)).

The combination screen with dactinomycin was performed essentially as described previously^13^. Briefly, the dactinomycin concentration resulting in 25% inhibition of metabolic activity (IC_25_, 10 nM) was calculated from the dactinomycin response curve in the single drug screen. Using a Tecan D300 drug printer, 10 nM dactinomycin was dispensed on top of the library in ready-to-use plates. All drugs were tested as single agents and combined with dactinomycin within the same plate (one replicate per plate); identical copies of all plates were used as a second replicate. The treatment effect of single and combination screens was quantified 72 h after cell seeding as metabolic activity readout using CellTiter-Glo® 2.0 (Promega, Madison, USA).

For more details on “drug screening and metabolic activity assay”, see Supplementary Note 5. Drug sensitivity scores (DSS_asym_) and dcDSSasym (= DSSasym (combo) – DSSasym (mono)), quantiles, percent inhibition at c_max_ and the IC_25_ concentration of dactinomycin were calculated using an in-house automated analysis pipeline based on ElHarouni et al.^13^. The response in relation to the cohort was determined as quantiles (percentage of samples with lower DSSasym compared to sample of interest, calculated for each drug of the library) to account for the skew-normal distribution of the DSSasym. The data analysis also includes the interpretation of the data on a 4-eyes principle and discussion of the data in our internal drug screening data review board meeting.

To monitor the three-dimensional growth of patient-derived cells from different diagnoses, transmitted light images of 384-well plates (wells B2-O23 of all three plates) were acquired with an ImageXpress Micro Confocal imaging system (Molecular Devices, San Jose, CA, USA) using a 20X Plan Apo objective (one field per well) immediately before performing the metabolic activity readout.

### Statistical information

All statistical analyses were performed with the software program R (R version 4.1.0; The R Foundation for Statistical Computing). Statistical tests for Pearson correlation were performed using the cor.test and the method = “pearson”. For Bland-Altman plots, the package “blandr”, version 0.5.1, was used. Heatmaps (unsupervised hierarchical clustering) were calculated with the packages pheatmap_1.0.12, dplyr_1.0.9, and readxl_1.4.1.

### Written informed consent statement and ethical approval

The study was conducted in accordance with Good Clinical Practice guidelines and the Declaration of Helsinki. All patients, their legally acceptable representatives, or both (if possible) provided written informed consent. Approval for the study protocol (and any modifications thereof) was obtained from independent ethics committees and the institutional review board at each participating center. The study was registered with the German Clinical Trial Register, number DRKS00007623.

### Reporting summary

Further information on research design is available in the Nature Research Reporting Summary linked to this article.

## Supplementary information

Supplementary Data

Supplementary Information

REPORTING SUMMARY

## Data Availability

Data are available as Supplementary Data and upon request. Detailed SOPs are available as Supplementary Notes. WES, lcWGS, RNA-sequencing, and methylation data generated by this study are available from the European Genome Archive, accession number EGAS00001005112. EGA requires controlled access, there is a corresponding Data Access Committee (DAC) to determine access permissions. Access to actual data files is not managed by the EGA.

## Code availability

Codes are available on GitHub: https://github.com/iTReX-Shiny/iTReX; https://github.com/KiTZ-Heidelberg/INFORM-TDSU-Manuscript.
