# Supplementary material for: Drug sensitivity profiling of 3D tumor tissue cultures in the pediatric precision oncology program INFORM
Source: NPJ Precis Oncol. 2022 Dec 27;6:94. doi: 10.1038/s41698-022-00335-y (PMC9794727; doi:10.1038/s41698-022-00335-y)
Supplement: Supplementary file 2 — Supplementary Information [file 41698_2022_335_MOESM2_ESM.pdf]

Drug sensitivity profiling of 3D tumor tissue cultures in the pediatric precision oncology program INFORM

Heike Peterziel[1,2,3], Nora Jamaladdin[1,2,3,4], Dina ElHarouni[1,4,5,6], Xenia F. Gerloff[1,2,3,7], Sonja Herter[1,2,3,4], Petra Fiesel[1,8], Yannick Berker[1,2,3], Mirjam Blattner-Johnson[1,9], Kathrin Schramm[1,9], Barbara C. Jones[1,9,10], David Reuss[11], Laura Turunen[12], Aileen Friedenauer[1,2,3], Tim Holland-Letz[13], Martin Sill[1,6], Lena Weiser[14], Christopher Previti[1,6,14], Gnanaprakash Balasubramanian[1,6], Nicolas U. Gerber[15], Johannes Gojo[16], Caroline Hutter[17], Ingrid Øra[18], Olli Lohi[19], Antonis Kattamis[20], Bram de Wilde[21], Frank Westermann[1,22], Stephan Tippelt[23], Norbert Graf[24], Michaela Nathrath[25,26], Monika Sparber-Sauer[27,28], Astrid Sehested[29], Christof M. Kramm[30], Uta Dirksen[31], Olli Kallioniemi[12,32], Stefan M. Pfister[1,6,10], Cornelis M. van Tilburg[1,2,3,10], David T.W. Jones [1,9], Jani Saarela[12], Vilja Pietiäinen[12,32], Natalie Jäger[1,6], Matthias Schlesner[5,33], Annette Kopp-Schneider[13], Sina Oppermann[1,2,3], Till Milde[1,2,3,10], Olaf Witt[1,2,3,10]*, Ina Oehme[1,2,3]*#

Affiliations:

[1] Hopp Children's Cancer Center Heidelberg (KiTZ), 69120 Heidelberg, Germany
[2] Clinical Cooperation Unit Pediatric Oncology, German Cancer Research Center (DKFZ) and German Cancer Consortium (DKTK), 69120 Heidelberg, Germany
[3] National Center for Tumor Diseases (NCT), 69120 Heidelberg, Germany
[4] Faculty of Biosciences, Heidelberg University, 69120 Heidelberg, Germany
[5] Bioinformatics and Omics Data Analytics, German Cancer Research Center (DKFZ), 69120 Heidelberg, Germany
[6] Division of Pediatric Neurooncology, German Cancer Research Center (DKFZ) and German Cancer Consortium (DKTK), 69120 Heidelberg, Germany
[7] Faculty of Mathematics and Computer Science, Heidelberg University, 69120 Heidelberg, Germany
[7] Clinical Cooperation Unit Neuropathology, German Cancer Research Center (DKFZ), 69120 Heidelberg, Germany
[9] Division of Pediatric Glioma Research, German Cancer Research Center (DKFZ) and German Cancer Consortium (DKTK), 69120 Heidelberg, Germany
[10] Department of Pediatric Oncology, Hematology, Immunology and Pulmonology, Heidelberg University Hospital, 69120 Heidelberg, Germany
[11] Department Neuropathology at Institute of Pathology, Heidelberg University Hospital, 69120 Heidelberg, Germany
[12] Institute for Molecular Medicine Finland (FIMM), Helsinki Institute of Life Science (HiLIFE), University of Helsinki, 00014 Helsinki, Finland
[13] Division of Biostatistics, German Cancer Research Center (DKFZ), Heidelberg, Germany
[14] Core Facility Omics IT and Data Management (ODCF), German Cancer Research Center (DKFZ), 69120 Heidelberg, Germany
[15] Department of Oncology, University Children's Hospital Zürich, CH-8032 Zürich
[16] Department of Pediatrics and Adolescent Medicine, Comprehensive Cancer Center and Comprehensive Center for Pediatrics, Medical University of Vienna, 1090, Vienna, Austria
[17] St. Anna Children's Hospital, Department of Pediatrics and Adolescent Medicine, Medical University of Vienna, 1090 Vienna, Austria, and St. Anna Children's Cancer Research Institute (CCRI), Vienna, Austria
[18] Children's Hospital, Pediatric Oncology, Skåne University Hospital, Lund & Karolinska University Hospital, Stockholm, Sweden
[19] Faculty of Medicine and Health Technology, Tampere Center for Child Health Research, Tampere University, Tampere, Finland, and Tays Cancer Center, Tampere University Hospital, Tampere, Finland
[20] First Department of Pediatrics, National and Kapodistrian University of Athens, Athens, Greece
[21] Center for Medical Genetics, Ghent University, Ghent, Belgium
[22] Division of Neuroblastoma Genomics, German Cancer Research Center, Heidelberg, Germany
[23] Pediatrics III, Pediatric Oncology and Hematology, University Hospital Essen, Essen, Germany
[24] Department of Pediatric Oncology, Saarland University Medical Center, 66421 Homburg, Germany
[25] Department of Pediatric Oncology, Klinikum Kassel, Kassel, Germany
[26] Department of Pediatrics and Children's Cancer Research Center, Klinikum rechts der Isar, Technical University of Munich, Munich, Germany
[27] Klinikum der Landeshauptstadt Stuttgart gKAöR, Olgahospital, Stuttgart Cancer Center, Zentrum für Kinder-, Jugend- und Frauenmedizin, Pädiatrie 5 (Pädiatrische Onkologie, Hämatologie, Immunologie), Stuttgart, Germany
[28] University of Medicine Tübingen, Tübingen, Germany
[29] Department of Pediatrics and Adolescent Medicine, Rigshospitalet, Copenhagen, Denmark
[30] Division of Pediatric Hematology and Oncology, University Medical Center Göttingen, Göttingen, Germany
[31] Pediatrics III Pediatric Hematology, Oncology, Immunology, Cardiology, Pulmonology, West German Cancer Center, University Hospital Essen, Essen, Germany
[32] iCAN Digital Precision Cancer Medicine Flagship, University of Helsinki, Helsinki, FI-00014, Finland
[33] Biomedical Informatics, Data Mining and Data Analytics, Faculty of Applied Computer Science and Medical Faculty, University of Augsburg, Augsburg, Germany

* these authors contributed equally
# correspondence: i.oehme@kitz-heidelberg.de

Supplementary Figure and Legends

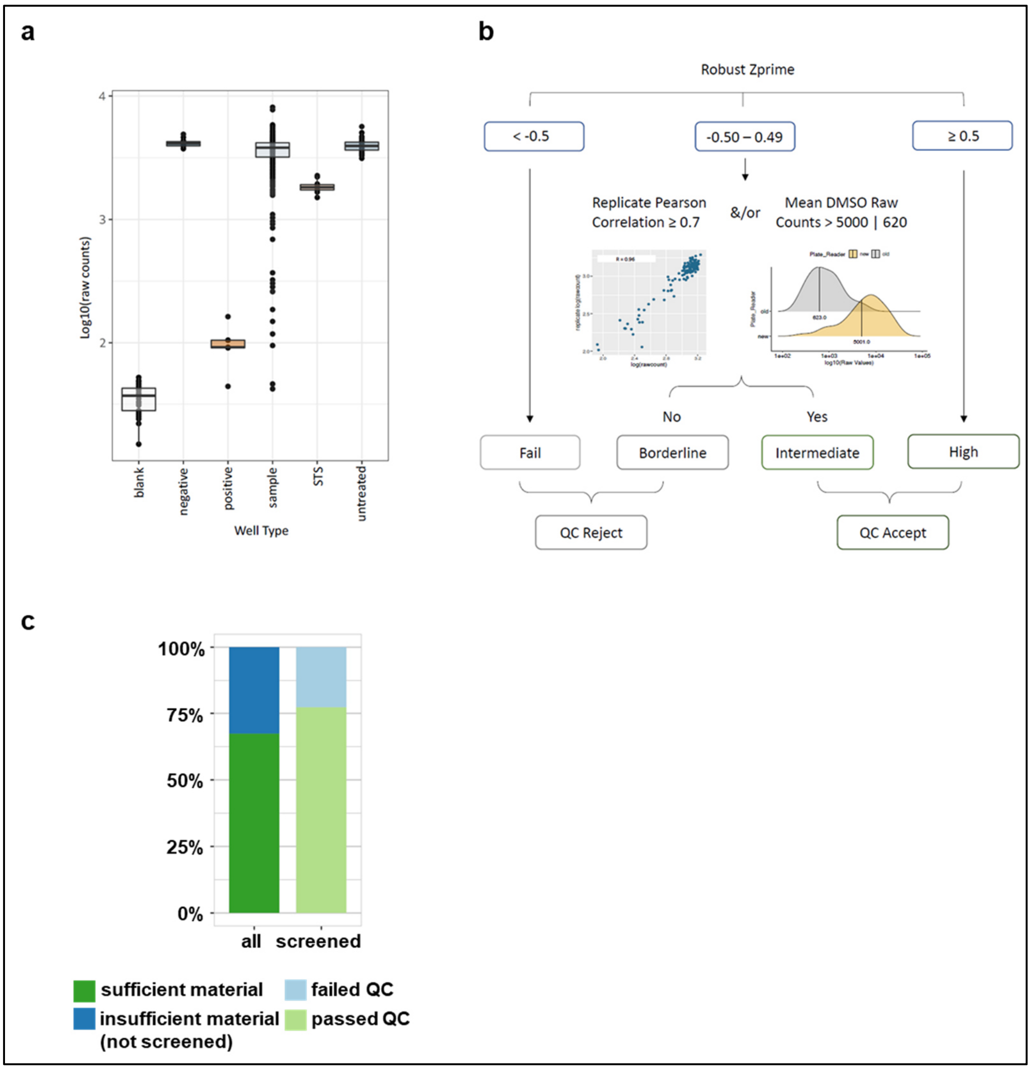

Supplementary Figure 1: Quality control classification criteria. a) Boxplot quality control output demonstrating the log10 raw count distribution of each well type screen on the profiled plate. Box plots reveal the median distribution per well type, while the outlined boxplot frame represent the 25% and 75% distribution of the readcounts per well type. The annotated dots represent each well measurement within the analyzed plate. The quality control Z' (Zprime) is assessed based on the measured separation of controls (positive and negative controls). A robust Zprime is calculated based on the dispersion of the control mad (median absolute deviation) and the control median, which is less sensitive to outliers.

$$Z' = 1 - \frac{3 * (mad(pos) + mad(neg))}{abs(median(pos) - median(neg))}$$

Negative (neg): DMSO-treated cells; positive (pos): cells treated with 100 µM benzethonium chloride; sample: drug-treated cells; STS: cells treated with 250 nM staurosporine (sublethal concentration of staurosporine to additionally control for plate-specific effects); blank: empty wells; untreated: cells without any treatment. **b)** The robust Zprime is used as a primary quantitative measure for QC criteria classification. A robust Zprime of less than -0.5 indicates a QC failed screen, more than or equal to 0.5 indicates a high QC screen, while samples lying between -0.5 and 0.49 must further pass a second step of QC filtering. In the latter case, a replicate Pearson correlation between drug replicates of 0.7 and higher and/or a mean raw count more than 30% quantile of the plate reader output for the negative control (DMSO) [5000 for the PheraStar/Tecan Spark plate reader and 620 for the Optima plate reader] is regarded as an intermediate QC. Only intermediate and high QC classifications are considered QC Accepted in the study. **c)** Bar diagram displaying the percentages of sufficient/insufficient viable material, and passed/failed QCs.

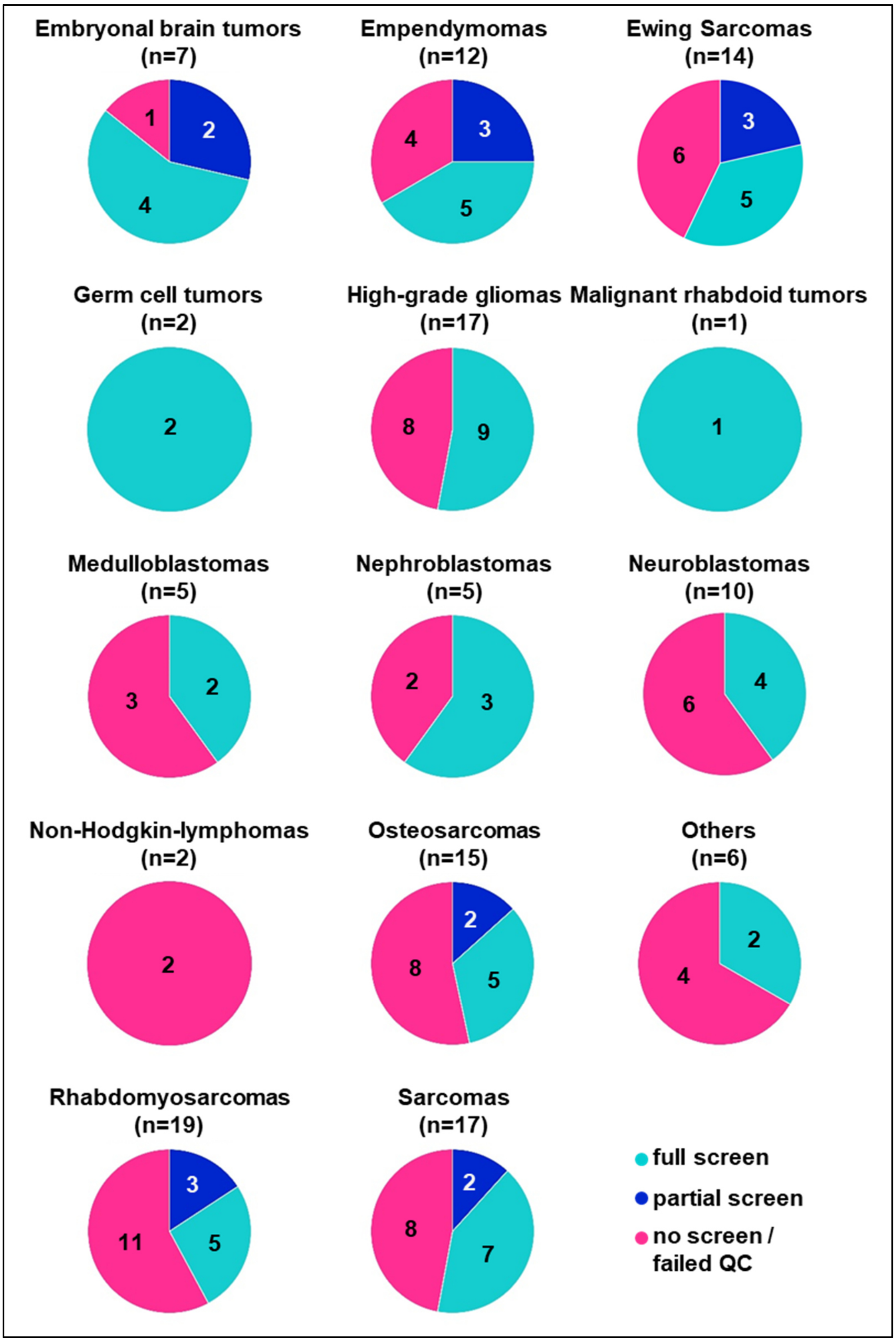

Supplementary Figure 2: Pie charts, divided into the different diagnoses, reflecting the absolute numbers for screen types for all samples accepted during the DSP pilot phase. Full screens were performed with the complete library consisting of three plates; partial screen stands for screens with one or two plates in the case of limited patient material. Samples that could not be screened or with the screen failing QC are combined (no screen/failed QC). The color code is shown at the bottom-right.

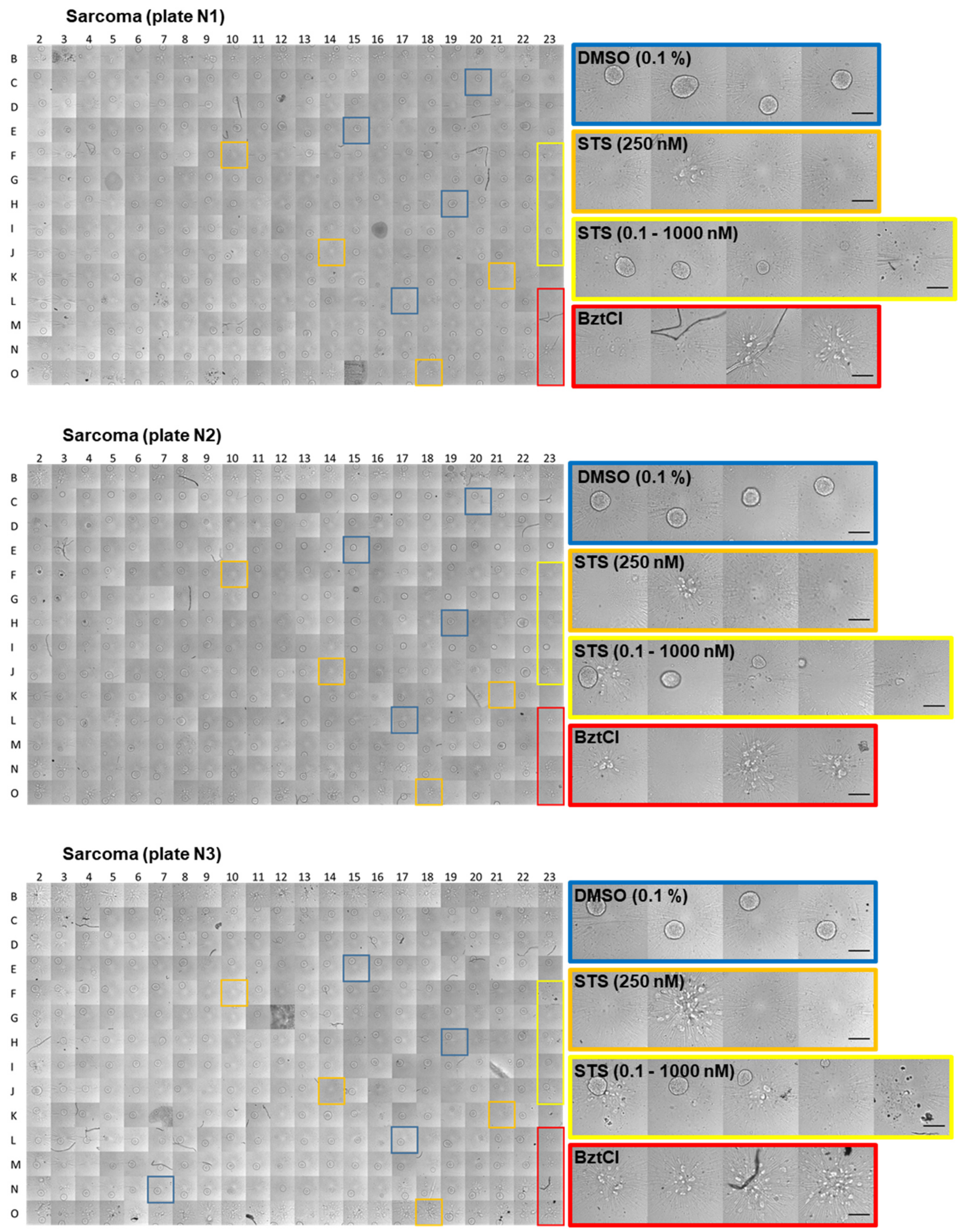

Supplementary Figure 3: Bright-field images illustrating three-dimensional growth of cells from a sarcoma sample in 384-well assay plates. Images were taken 72 h after cell seeding. Wells B2-O23 of plates N1, N2 and N3, composing a full library plate set, are shown on the left. Note: The outer rows (A and P) and columns (1 and 24) were not imaged due to technical reasons (outer well autofocus problems). Exemplary wells for the different assay controls, for which magnifications are displayed on the right side of the corresponding plate, are highlighted by different colors. DMSO: Negative control wells containing 0.1% DMSO (blue); STS (250 nM; 0.1-1000 nM): Wells preprinted with a fixed concentration of staurosporine (250 nM, orange) and with a staurosporine concentration range (0.1, 1, 10, 100 and 1000 nM; yellow). BztCl: Wells with 100 µM benzethonium chloride (red). Scale bar: 200 µm.

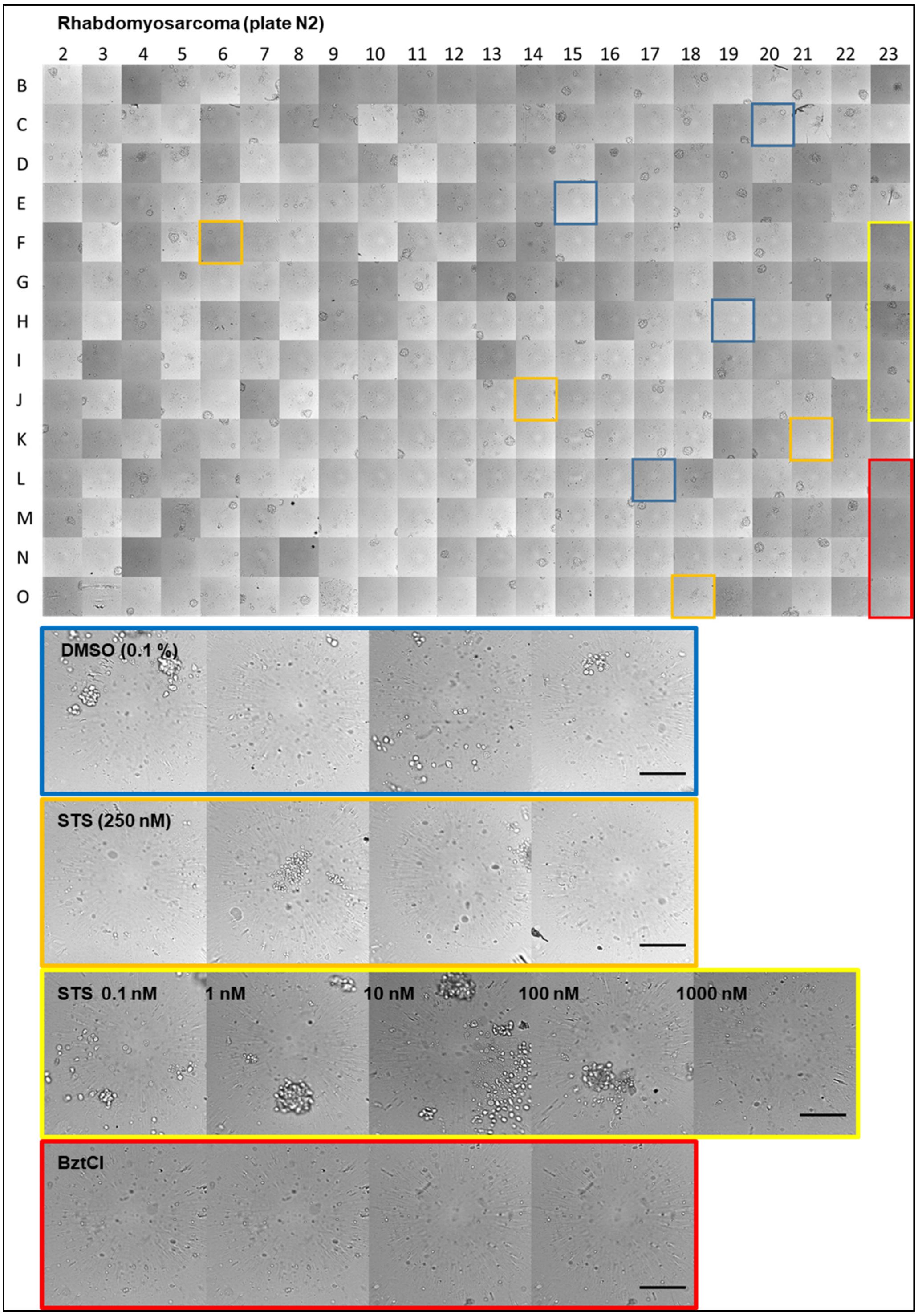

Supplementary Figure 4: Bright-field images taken 72 h after seeding cells from a rhabdomyosarcoma sample. Plate N2 (top) and corresponding magnifications of assay control wells (bottom panel) are shown. For details, see Supplementary Figure 3. Scale bar: 200 µm.

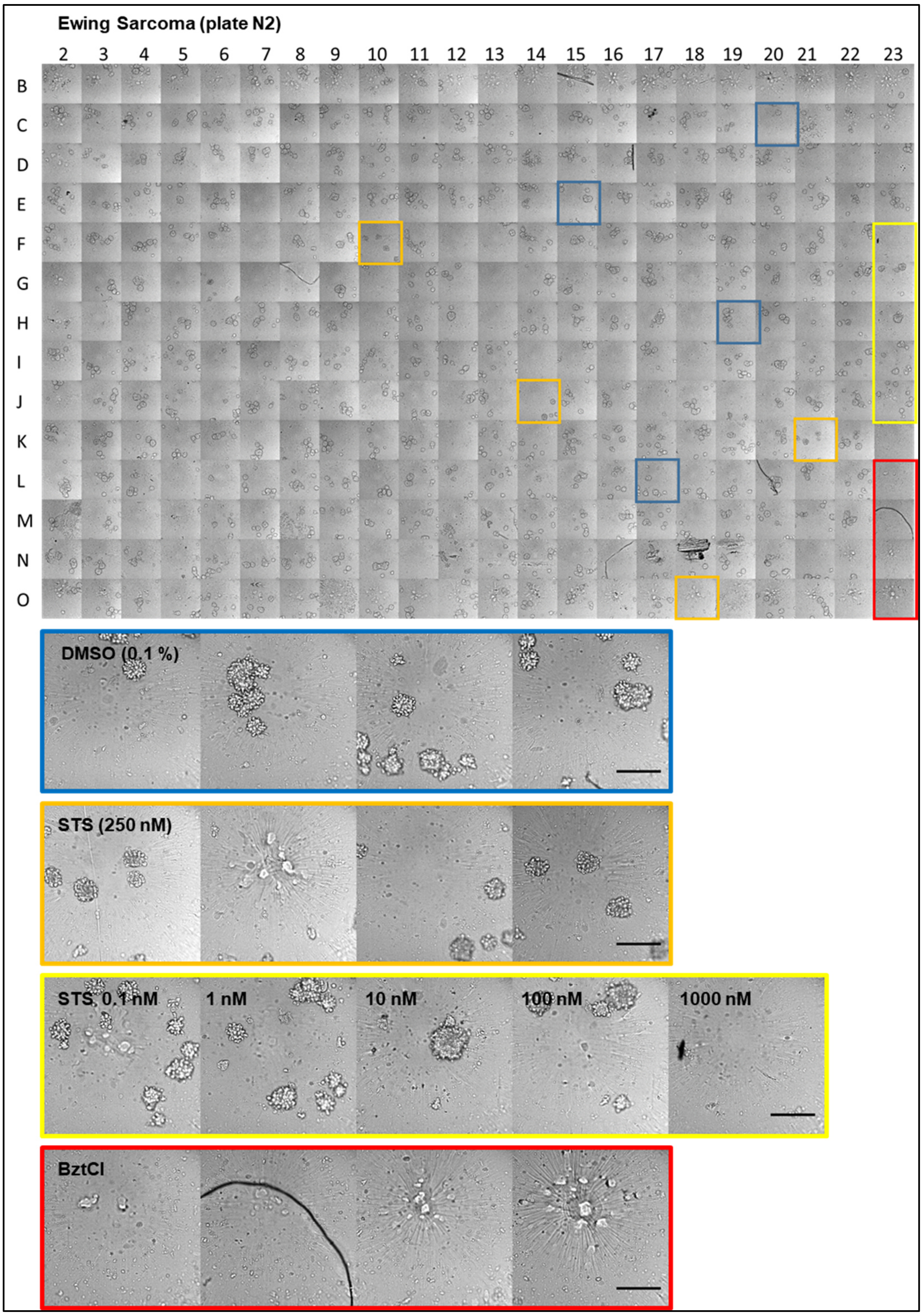

Supplementary Figure 5: Bright-field images taken 72 h after seeding cells isolated from an Ewing sarcoma sample. Plate N2 (top) and corresponding magnifications of assay control wells (bottom panel) are shown. For details, see Supplementary Figure 3. Scale bar: 200 µm.

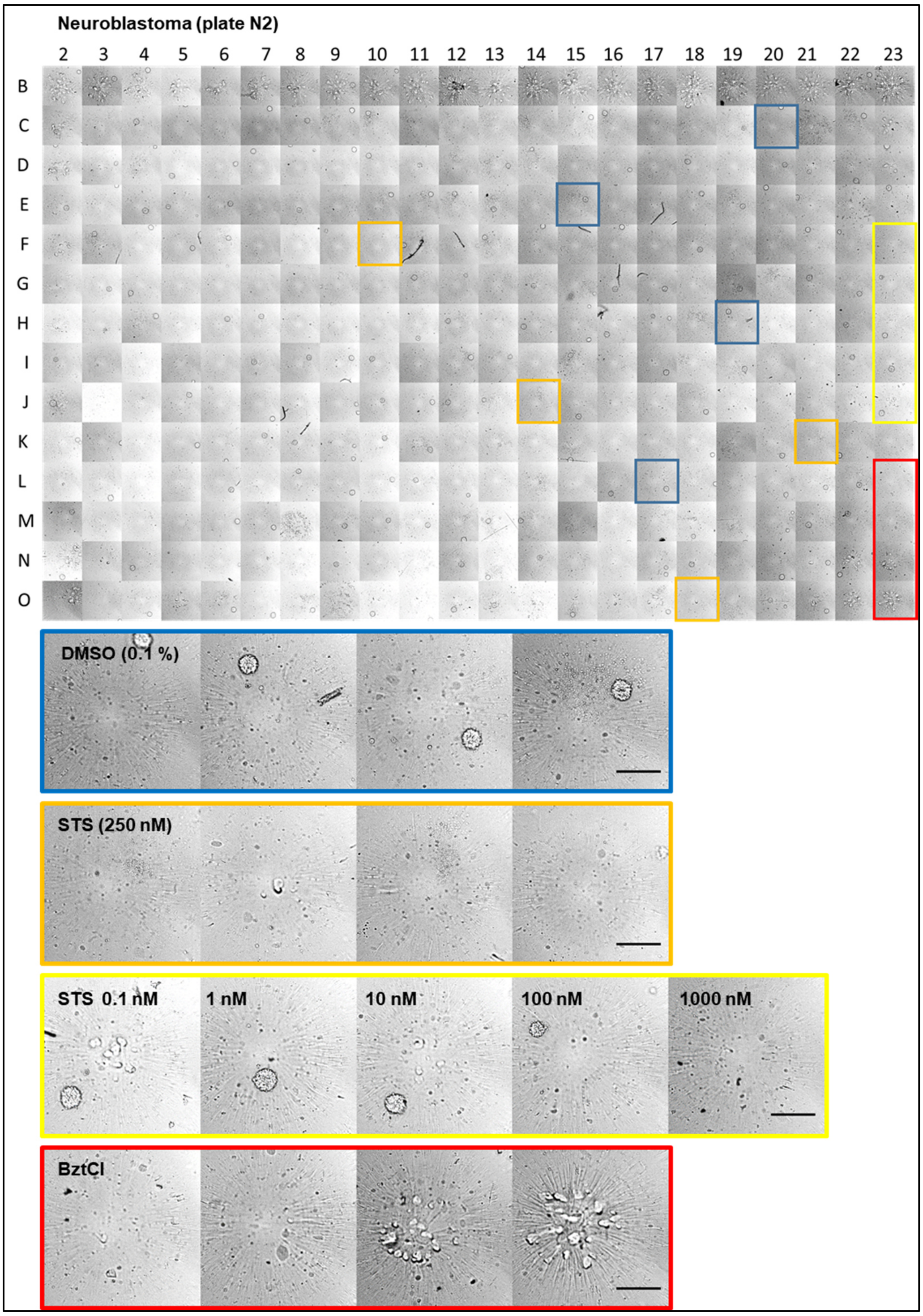

Supplementary Figure 6: Bright-field images taken 72 h after seeding cells isolated from a neuroblastoma sample. Plate N2 (top) and corresponding magnifications of assay control wells (bottom panel) are shown. For details, see Supplementary Figure 3. Scale bar: 200 µm.

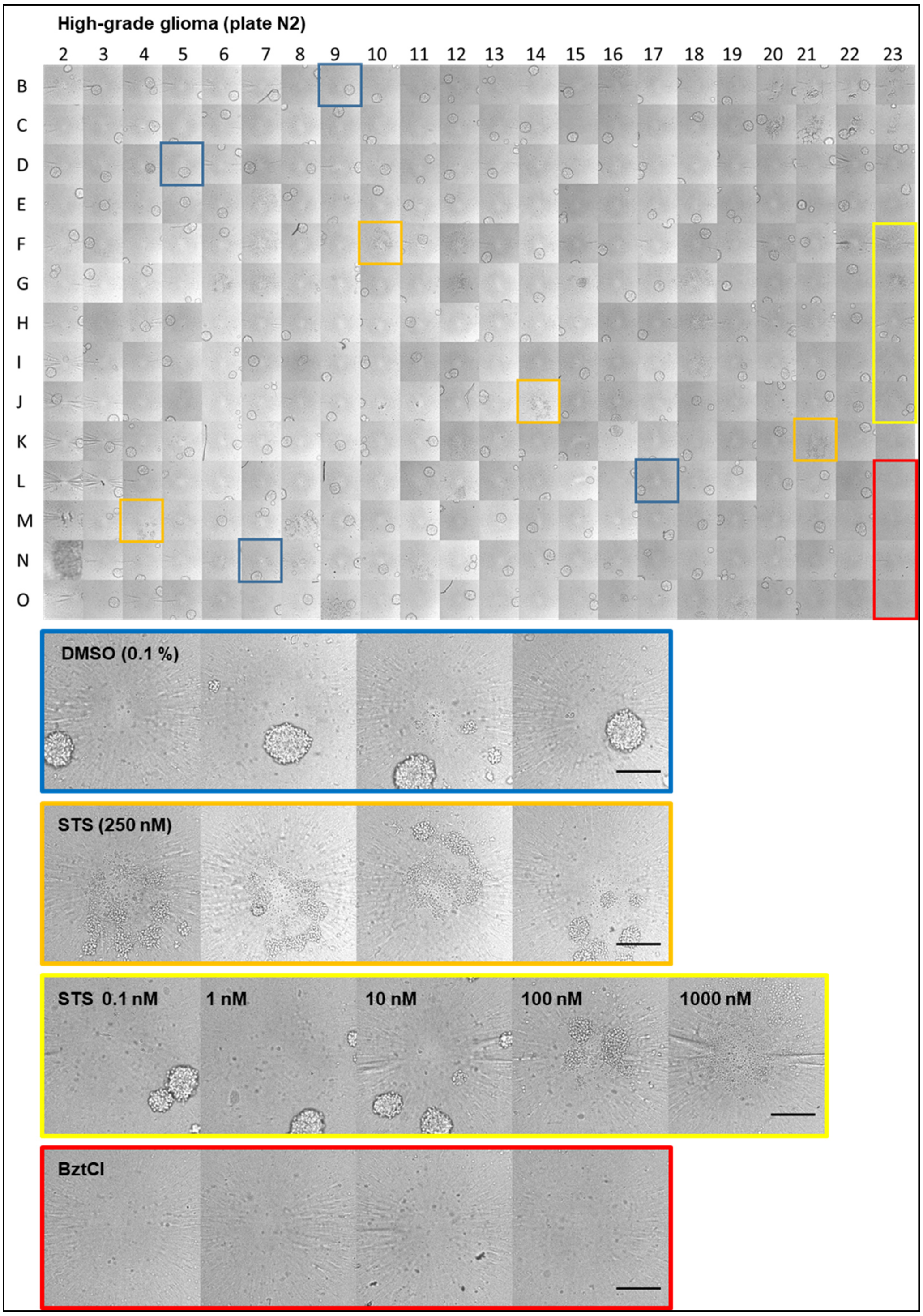

Supplementary Figure 7: Bright-field images taken 72 h after seeding cells isolated from a high-grade glioma sample. Plate N2 (top) and corresponding magnifications of assay control wells (bottom panel) are shown. For details, see Supplementary Figure 3. Scale bar: 200 µm.

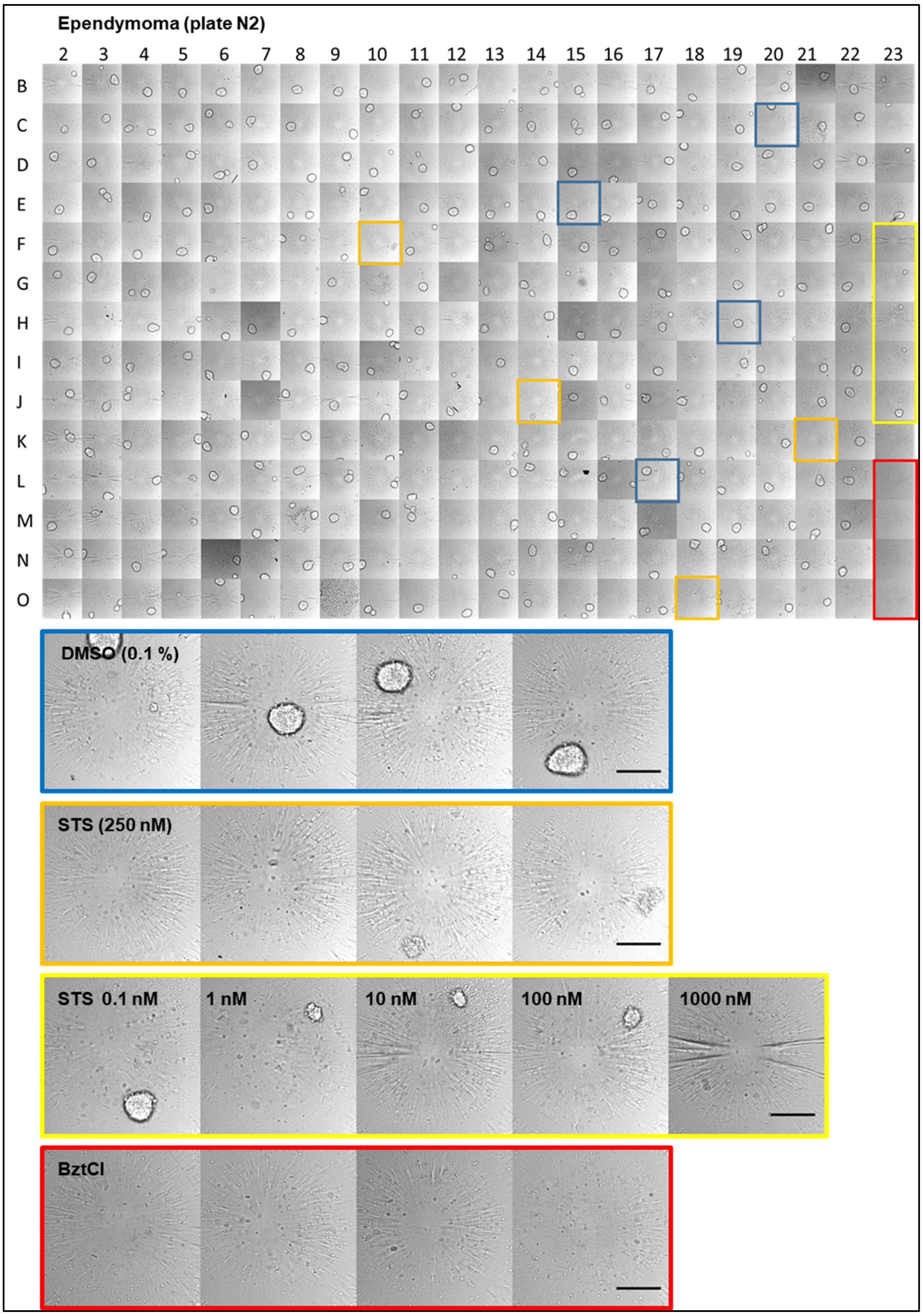

Supplementary Figure 8: Bright-field images taken 72 h after seeding cells isolated from an ependymoma sample. Plate N2 (top) and corresponding magnifications of assay control wells (bottom panel) are shown. For details, see Supplementary Figure 3. Scale bar: 200 µm.

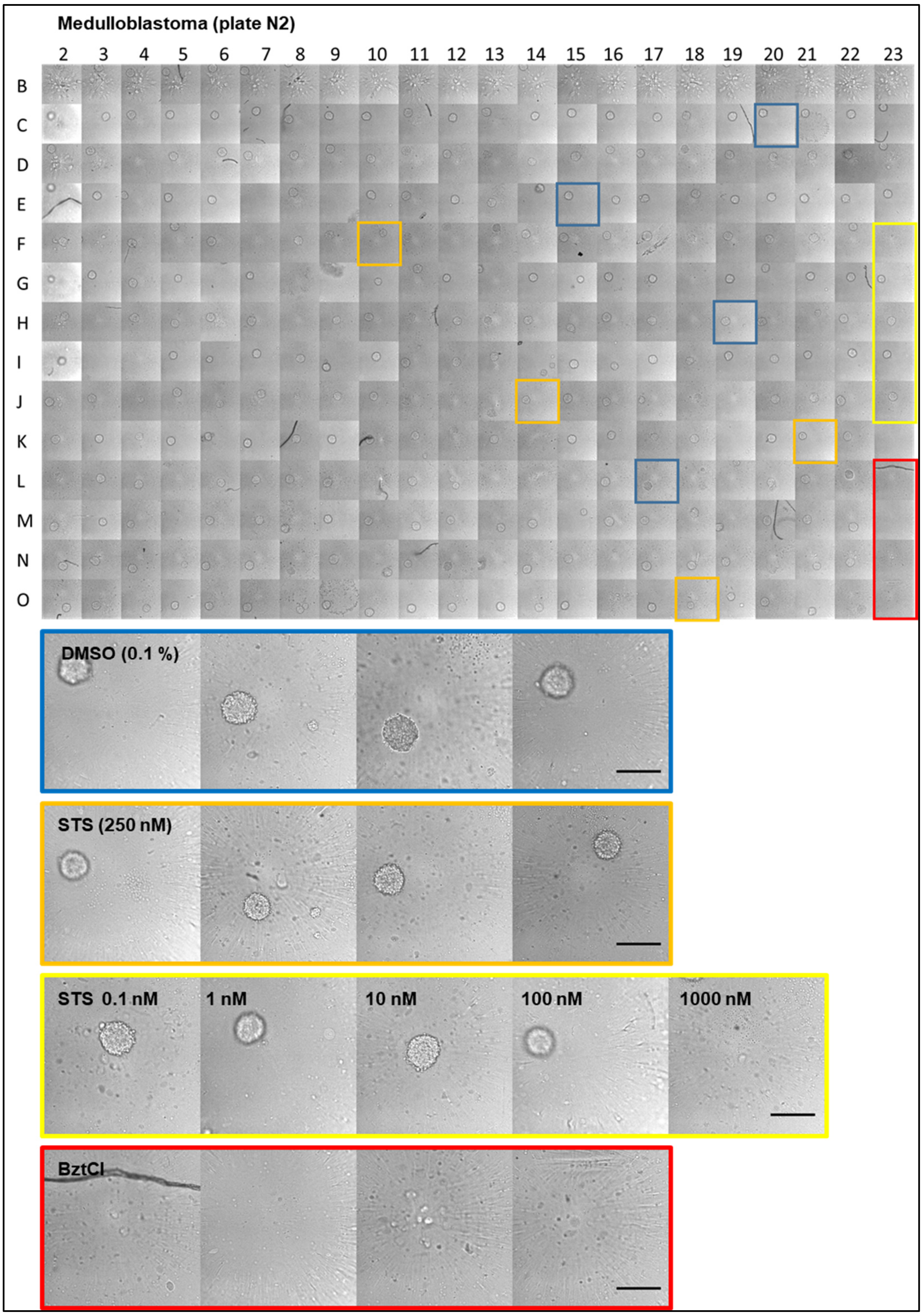

Supplementary Figure 9: Bright-field images taken 72 h after seeding cells isolated from a medulloblastoma sample. Plate N2 (top) and corresponding magnifications of assay control wells (bottom panel) are shown. For details, see Supplementary Figure 3. Scale bar: 200 µm.

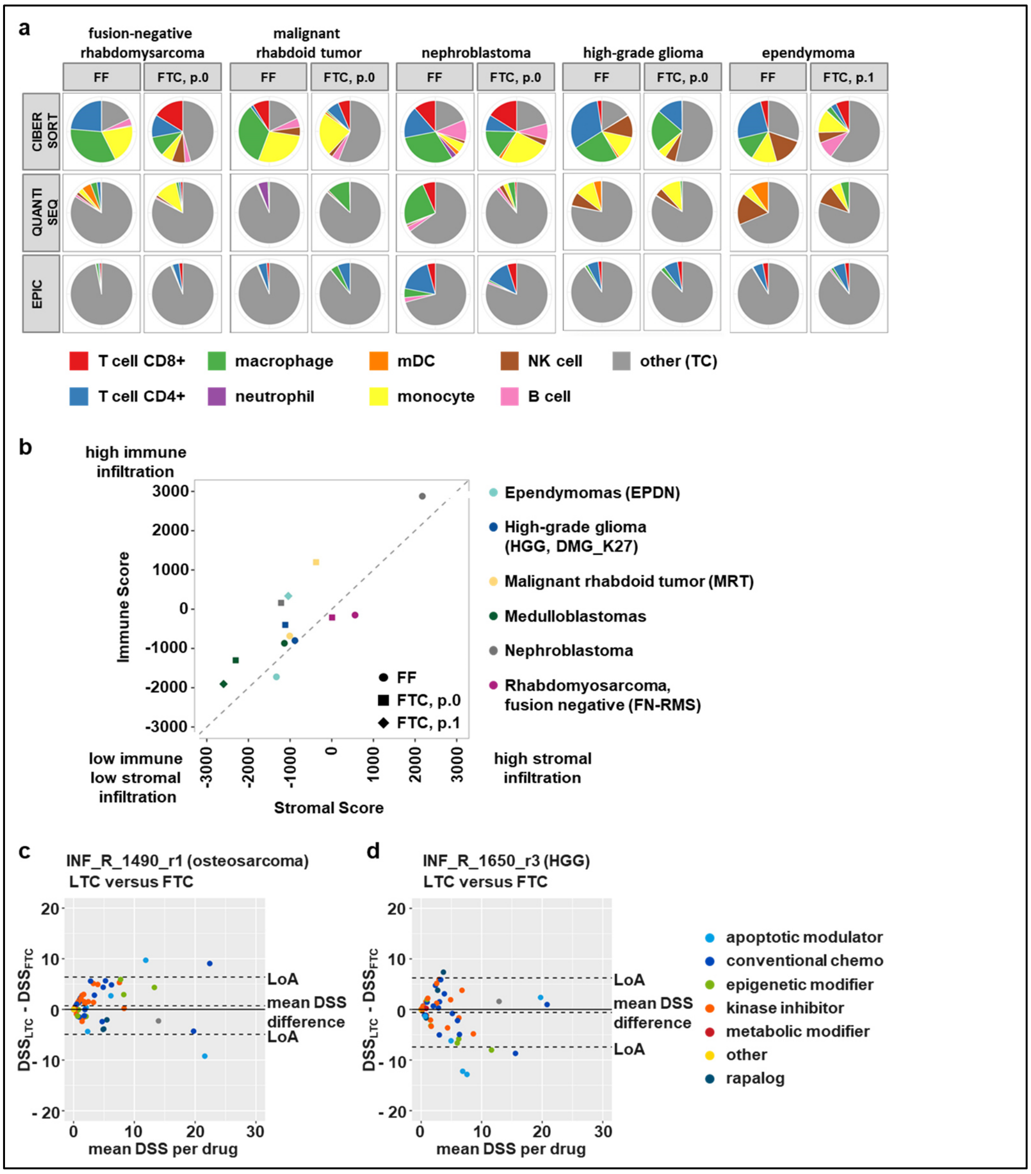

Supplementary Figure 10: Multicellular composition and comparison of FTC and matched LTC drug sensitivity. a) Immune cell type deconvolution results per sample pair with different (the most commonly used bulk RNA-seq deconvolution tools: CIBERSORT, QuantiSeq, and EPIC. FF, fresh frozen (original tumor); FTC p.0, fresh tissue culture directly after sample dissociation, passage 0. FTC, p.1, fresh tissue culture at the time point of drug screen seeding (passage 1). TC, tumor cell. **b)** ESTIMATE stromal and immune scores based on tumor bulk RNA-seq data. ESTIMATE provides scores for tumor purity, the level of stromal cells present (x-axis), and the infiltration level of immune cells (y-axis) in tumor tissues based on RNA-seq data. Higher scores correspond to higher infiltration of stromal/immune cells. FF, fresh frozen (original tumor); FTC p.0, fresh tissue culture directly after sample dissociation, passage 0. FTC, p.1, fresh tissue culture at the time point of drug screen seeding (passage 1). **c-d)** Bland-Altman plots for comparison of drug sensitivity scores of each drug for LTC versus FTC. The difference between the DSS_{asym} is plotted (y-axis) against the average (mean) DSS_{asym} for each drug. The color code on the right reflects the different functional drug classes. Cultures were derived from patient samples INF_R_1490_r1 (**c**; osteosarcoma) and INF_R_1650_r3 (**d**; HGG). FTC: fresh tissue culture of the original tumor; LTC: long-term culture. LoA: limits of agreement. DSS: drug sensitivity score (= DSS_{asym}).

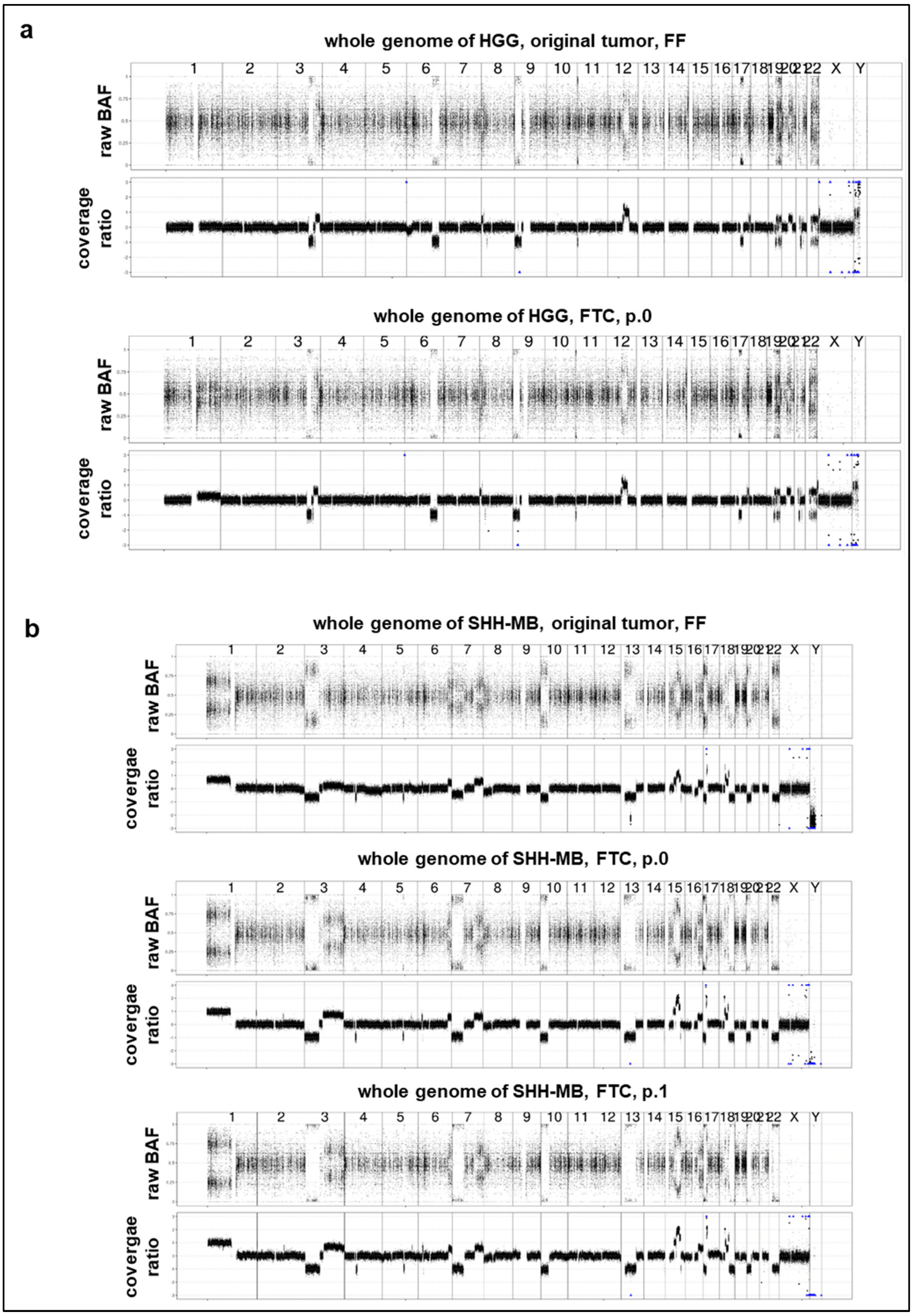

Supplementary Figure 11: Tumor copy number profile based on WES and lcWGS data. a) High-grade glioma (HGG) with known PATZ1-MN1 fusion. Enrichment of tumor cells is visible through a larger spread of raw BAF. **b)** SHH-medulloblastoma (MB) with a known germline TP53 point mutation (Li-Fraumeni patient). BAF, b-allele frequency. FF, fresh frozen (original tumor); FTC p.0, fresh tissue culture directly after sample dissociation, passage 0. FTC, p.1, fresh tissue culture at the time point of drug screen seeding (passage 1).

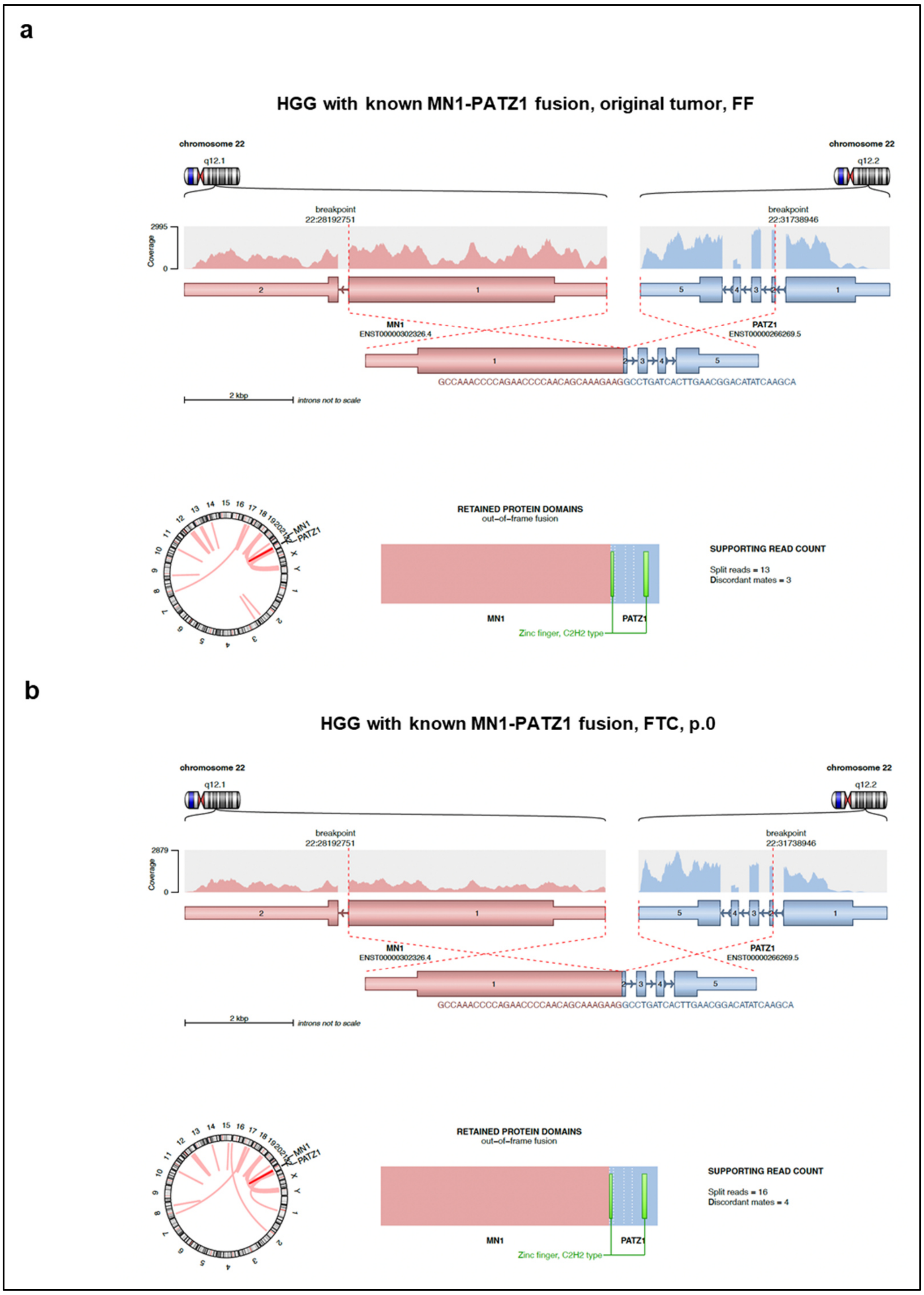

Supplementary Figure 12: Detection of gene fusions based on RNA-seq data with the tool "arriba". a) High-grade glioma (HGG) with known PATZ1-MN1 fusion, original tumor, FF (fresh frozen). **b)** High-grade glioma (HGG) with known PATZ1-MN1 fusion, fresh tissue culture (FTC) directly after sample dissociation, passage 0.

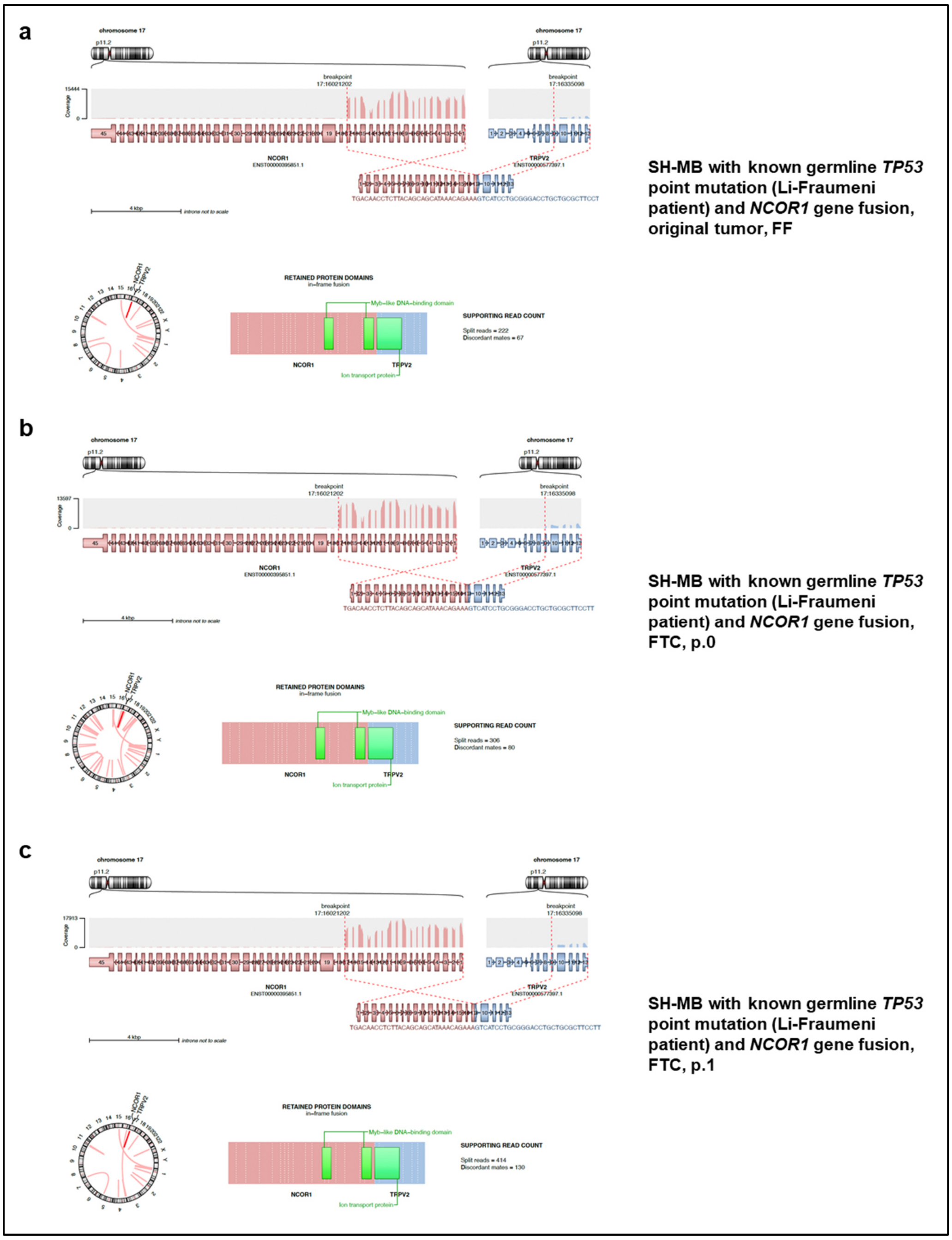

Supplementary Figure 13: Detection of gene fusions based on RNA-seq data with the tool "arriba". a) SHH-medulloblastoma (MB) with a known germline TP53 point mutation (Li-Fraumeni patient) and *NCOR1* gene fusion, original tumor, FF (fresh frozen). **b)** SHH-medulloblastoma (MB) with known germline TP53 point mutation (Li-Fraumeni patient) and *NCOR1* gene fusion, fresh tissue culture (FTC) directly after sample dissociation, passage 0. **c)** SHH-medulloblastoma (MB) with known germline TP53 point mutation (Li-Fraumeni patient) and *NCOR1* gene fusion, fresh tissue culture (FTC) at the time point of drug screen seeding (passage 1).

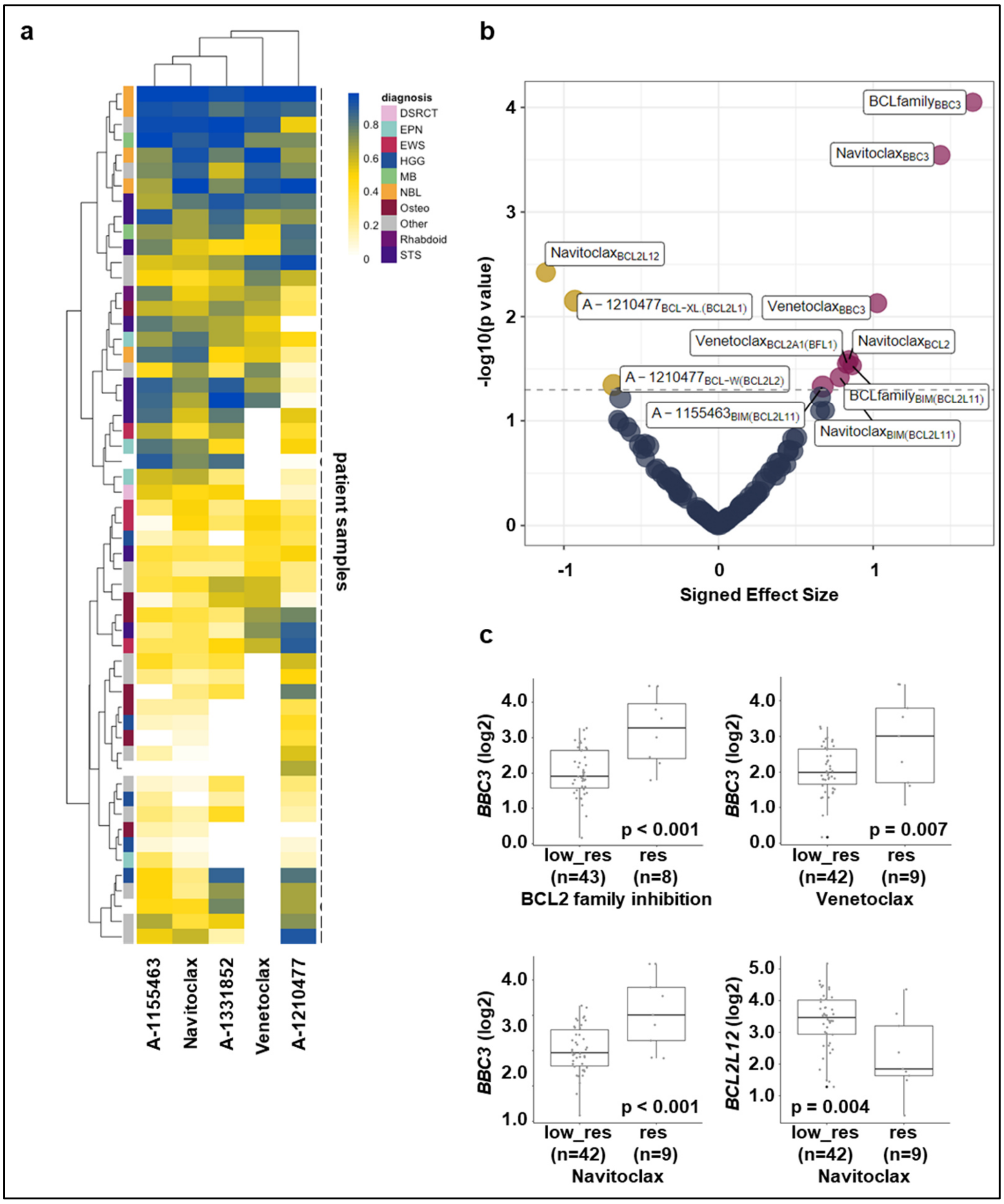

Supplementary Figure 14: Response to BCL2 family inhibition. a) Unsupervised hierarchical clustering based on DSS_{asym} quantile ranks for the five BCL2 family inhibitors included in the library. The color scale shown at the top represents quantile values. The diagnoses are highlighted with colored bars on the left side (color code is depicted on the top right). DSRCT: desmoplastic small round cell tumor; EPN: ependymoma; EWS: Ewing sarcoma; HGG: high-grade glioma; MB: medulloblastoma; NBL: neuroblastoma; Osteo: osteosarcoma; Rhabdoid: malignant rhabdoid tumor; STS: soft tissue sarcoma **b)** Volcano plot displaying Multivariate Analysis of Variance (MANOVA) associations between BCL2 family drug responses and BCL2 family expression (RNA-seq) genes. Samples with a quantile value of ≥ 0.75 (75%) were defined as responders, and samples with a quantile < 0.75 were defined as low responders. The x-axis shows the signed effect size (log fold change); the y-axis indicates the statistical significance (-log10 (p values); horizontal dashed line corresponds to $P = 0.05$). BCL2 family members with significantly higher (right arm) or lower (left arm) expression in responders are highlighted by purple and orange dots, respectively. BCL2 family member gene names and the associated library drugs are shown for these significantly differentially expressed genes. **c)** Box plots illustrating the log2 expression value of BCL2 family genes within the top reported associations across groups of low responders (low_res) and responders (res) for each of the top associated drugs (mean response of the BCL2 family, venetoclax, and navitoclax). All presented boxplots indicate the median log2 expression and the box edges span the 25-75% quartile range; whiskers extend the distribution spanning the 5-95% quartiles and outlier points are visualized beyond the denoted whiskers.

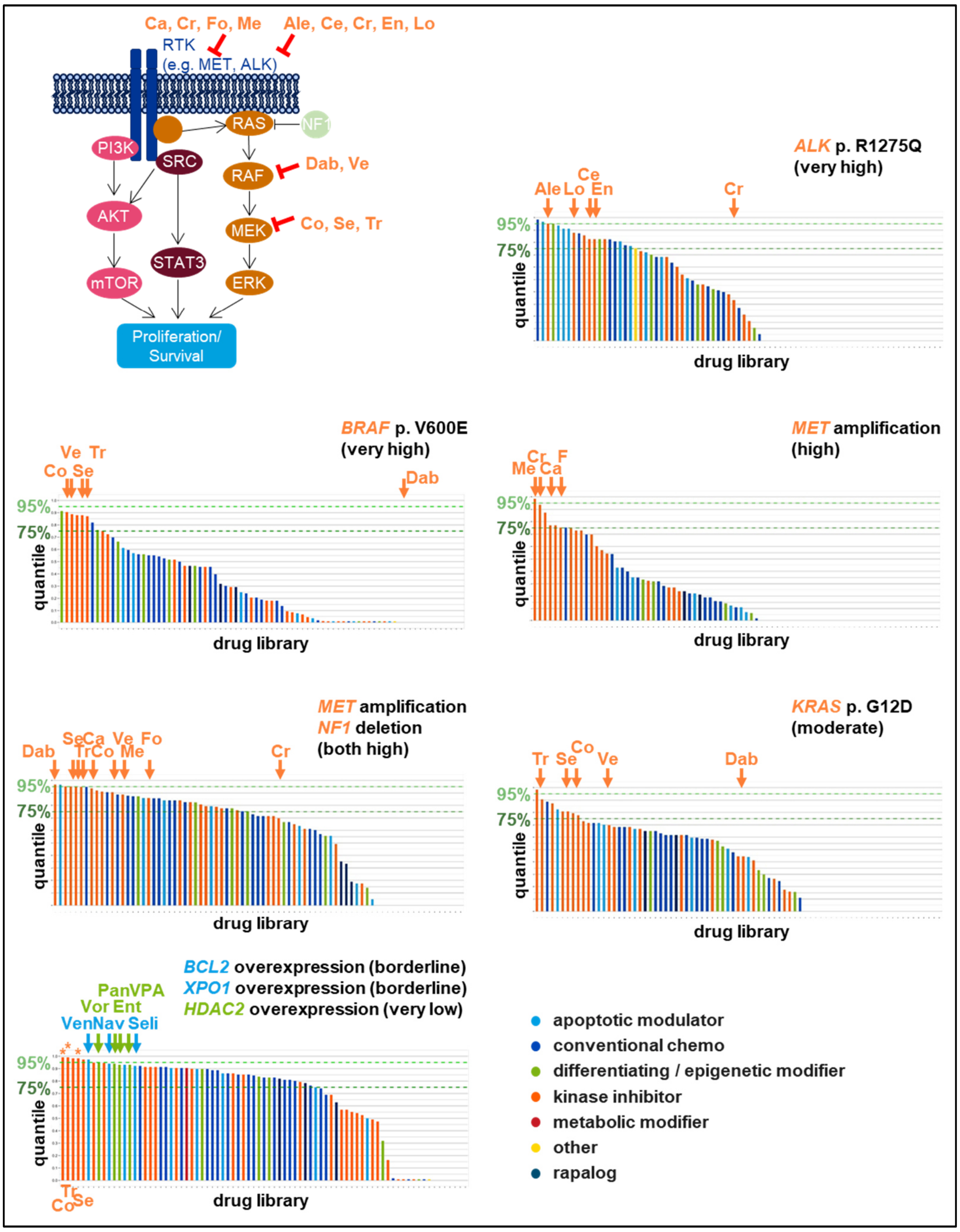

Supplementary Figure 15: Quantile waterfall plots of DSP results for exemplary samples with actionable molecular alterations identified by NGS. The schematic representation on the top right illustrates the signaling pathway components inhibited by the kinase inhibitors highlighted in the waterfall plots below. Molecular alterations (with colors reflecting the drug class of matching drugs) and evidence levels (as described for the INFORM registry in van Tilburg et al., 2021; PMID: 34373263) are named above each waterfall plot. Horizontal dashed lines indicate the 95% and 75% quantiles. The library drugs are color coded according to their drug class (color code on the bottom right). Quantile ranks for drugs within the library matching the respective molecular alteration are indicated by arrow bars and drug name abbreviations. * mark unexpected drug hits (MEK inhibitors). ALK inhibitors: Ale, Alectinib; Ce, Ceritinib; Cr, Crizotinib; En, Entrectinib; Lo, Lorlatinib. BCL2 family inhibitors: Nav, Navitoclax; Ven, Venetoclax. BRAF inhibitors: Ve, Vemurafenib; Dab, Dabrafenib. HDAC inhibitors: Ent, Entinostat; Pan, Panobinostat; VPA, Valproic Acid; Vor, Vorinostat. MEK inhibitors: Cobimetinib, Trametinib, Selumetinib. MET inhibitors: Ca, Cabozantinib; Cr, Crizotinib; F, Foretinib; Me, Merestinib. XPO inhibitor: Seli, Selinexor.

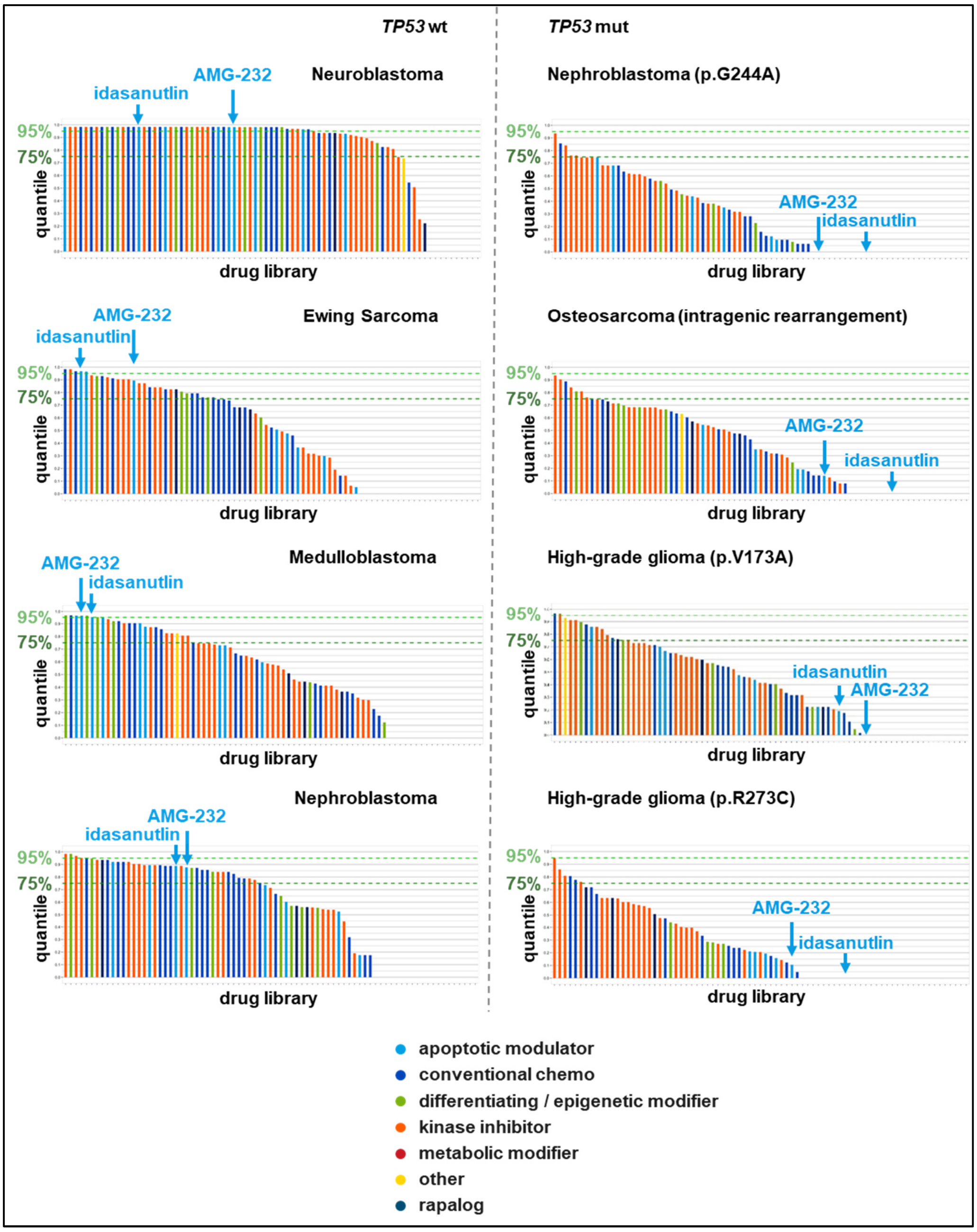

Supplementary Figure 16: Quantile ranks for Idasanutlin and AMG-232 for DSP of *TP53* wild-type and *TP53* mutant samples. Waterfall plots illustrating the DSS_{asym} quantiles of all library drugs for four *TP53* wild-type (*TP53* wt, left plots) and *TP53* mutants (*TP53* mut, right plots). Tumor diagnosis and type of *TP53* alteration are shown above each waterfall plot. Horizontal dashed lines indicate 95% and 75% quantiles; blue arrows mark idasanutlin and AMG-232 quantile ranks. The color code below the plots reflects the drug classes.

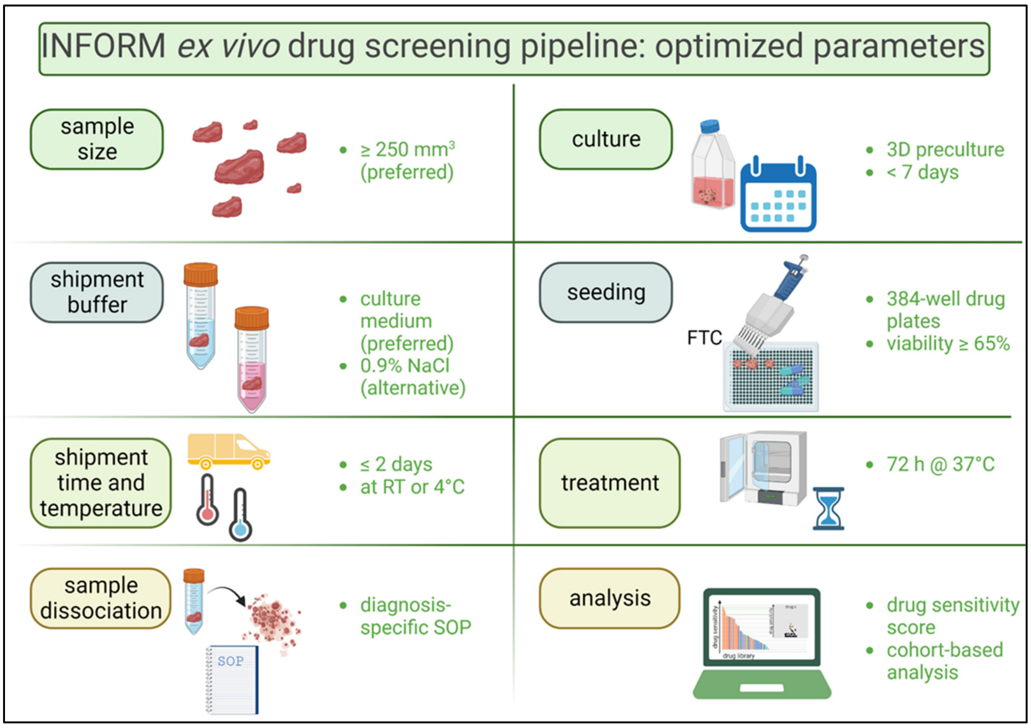

Supplementary Figure 17: Overview of optimized parameters of the INFORM *ex vivo* drug screening pipeline during the 2-year pilot phase. Sample collection and shipment, preferably piece sizes above 250 mm^3 (pea-size) and shipped in serum-free culture medium. Smaller tissue pieces do not per se exclude screening success. Similarly, if culture medium is not available, physiological saline solution can be used as an alternative. Sample dissociation with diagnosis-optimized protocols. Preculture preferable of maximal seven days before drug screening. Hit selection in consideration of drug sensitivity scores and a cohort-based analysis. Created with BioRender.com.

Supplementary Notes

Note 1: Shipment protocol

- Register the patient in the INFORM/MARVIN database and complete the registration form. Generate the shipment form in MARVIN by filling in all required information.
- Put vital tumor tissue in Falcon tubes filled with 0.9% NaCl or serum-free cell culture medium and do not freeze.
- Send at room temperature or cool (cool pack; do not freeze!) within 24 h post-surgery via overnight express together with the shipment form to the INFORM incoming lab as indicated. Optional: send 3-5 mL heparin blood (minimum leukocyte number ≥ 5000/µl) at room temperature as a viable normal control.
- Alert the INFORM team by sending an e-mail indicating the soon arrival of vital tumor tissue for drug sensitivity profiling.
- Submit fresh frozen tissue and blood samples separately for molecular diagnostics/target identification.

Note 2: Processing of vital tumor material

Prior to the pilot phase of INFORM *ex vivo* drug screening, the procedure for processing CNS tumor samples and brain metastases was established based on an adult brain tumor dissociation protocol [1]. Using, albeit limitedly, available pediatric brain tumor samples, the protocol was adapted for pediatric samples to account for the generally smaller sample sizes and lower cell yield compared to adult glioblastoma specimens. Optimization steps mainly involved 1) prolonged centrifugation after dissociation, 2) omission of centrifugation and red blood cell lysis steps for very small initial sample sizes, and 3) the use of a smaller cell culture format facilitating recovery, adaptation to serum-free conditions and formation of free-floating spheroids while also allowing for spontaneous cell adhesion, if applicable.
For non-brain tumor samples, there was no tumor material available to systematically test different dissociation protocols. Hence, we performed a comprehensive review of the literature, with the aim of defining conditions for the screening of patient-derived material within a clinically meaningful time, allowing the discussion of results in the tumor board, ideally within three to four weeks after surgery/biopsy. Criteria for the search included 1) high success rate of organoid generation or of patient-derived xenograft (PDX) engraftment from fresh tumor specimens, 2) description of short- and long-term cell culture generation for different adult or pediatric solid tumors, and 3) reports on approaches to screen mixed cell populations (tumor and non-tumor cells). We considered approaches described in Stewart et al.[2], Kodack et al.[3], and Pauli et al.[4] to develop diagnosis-specific protocols for neuroblastomas, osteosarcomas and soft tissue sarcomas (with the latter also used as default for other (rare) non-brain tumor diagnoses). All protocols were additionally modified to conform to serum-free conditions and a “one-fits all” culture and screening medium (TSM complete; based on Lin & Monje[5]). Throughout the pilot phase, details of the sample processing were individually adapted for each tumor specimen based on previous observations on samples with the same diagnoses or with similar appearance and size. The

limited size of patient-derived tissue samples did not allow a systematic comparison of several protocols with one and the same sample.
The materials listed are the products used specifically for the experiments described in this manuscript. Comparable products from other providers may be equally suitable for conducting the experiments.

Note 2.1: Soft tissue sarcomas

Note: We currently use this protocol also for rare (non-brain) tumor diagnoses (i.e., hepatoblastomas, nephroblastomas, rhabdoid tumors, mesotheliomas)

Materials:

DMEM (without supplements; cat. No. 41965-039; Gibco)
Trypsin (cat no. T9935, Sigma–Aldrich; stock solution 10 mg/mL, store at -80°C)
Collagenase II (cat. no. 17101015; Thermo Fisher Scientific; stock solution 50 mg/mL, store at -20°C)
Trypsin inhibitor (Sigma catalog no. T6522, stock solution 10 mg/mL, store at 4°C)
DNase (cat. no. D4513, Sigma–Aldrich; stock solution 2 mg/mL in 0.15 M NaCl)
M $MgCl_2$ (cat. no. AM9530G; Thermo Fisher Scientific)
ACK (ammonium-chloride-potassium) lysing buffer (cat. no. A1049201; Thermo Fisher Scientific)
10 cm cell culture dish
Sterile scalpels; scissors
50 mL conical tubes
70 or 100 µm Falcon® cell strainer (cat no. 431751/431752; Corning)
TSM base and TSM complete (for details see “Medium recipes”)

Procedure:

1) Transfer the tumor sample to a cell culture dish (without medium) and mince thoroughly using sterile scalpels or scissors
2) Add DMEM, transfer all tumor fragments to a 50 mL conical tube, and fill with DMEM to a final volume of 5 to 50 mL (depending on the size of the sample).
3) Add 12 µl trypsin (10 mg/mL) per mL (i.e., 240 µl to 20 mL DMEM) and 20 µl Collagenase II (50 mg/mL) per mL (i.e., 400 µl to 20 mL DMEM)
4) Incubate for 60 min at 37°C (water bath); mix every ~ 10 minutes by inverting the tube several times.
5) Add 12 µl trypsin inhibitor (10 mg/mL) per mL (i.e., 240 µl to 20 mL DMEM) to stop the enzymatic digestion.

6) Prepare a 1:1 mix of 1 M $MgCl_2$ and DNase (2 mg/mL) and repeatedly add 60 µl of the mix to the sample until the tumor fragments settle on the bottom of the tube (digestion of DNA released from dead cells); DNase is sensitive to mechanical stress (do not vortex, avoid excessive shear stress)
7) Filter through a 70 µm or, preferably, 100 µm cell strainer; spin down (400-500 g, 10 min, RT)
8) Resuspend the cell pellet in 2 mL ACK Lysing buffer (or more, depending on the size of the pellet) to lyse red blood cells and incubate for **2**-5 min at RT; fill up to 10 mL with TSM base and spin down the cells (400-500 g, 10 min RT)

Optional: repeat this step, if necessary (considerable red "ring" of erythrocytes in the pellet)

Note of caution: Omit red blood cell lysis, if the material is very limited (small cell pellet), or if there is no red "ring" of erythrocytes visible.

9) Resuspend the cell pellet in TSM complete (5-10 mL), count and seed according to the desired use. We cultured the cells on Corning® CellBIND® 6-well Clear Multiple Well Plates (cat no. 3335, Corning) for two to seven days prior to seeding on drug plates and to start the long-term cultures.

Note 2.2: Osteosarcomas

Materials:

PBS (-Ca/Mg; cat. no. 17-512F; Lonza) Trypsin (cat no. T9935, Sigma–Aldrich; stock solution 10 mg/mL, store at -80°C)
Collagenase II (cat. no. 17101015; Thermo Fisher Scientific; stock solution 50 mg/mL, store at -20°C)
Trypsin inhibitor (Sigma catalog no. T6522, stock solution 10 mg/mL, store at 4°C)
DNase (cat. no. D4513, Sigma–Aldrich; stock solution 2 mg/mL in 0.15 M NaCl; store at -20°C)
1M $MgCl_2$ (cat. no. AM9530G; Thermo Fisher Scientific)
ACK (ammonium-chloride-potassium) lysing buffer (cat. no. A1049201; Thermo Fisher Scientific)
10 cm cell culture dish
Sterile scalpels; scissors
100 mL glass bottle, magnet stir bar and magnetic stirrer/37°C waterbath, or 50 mL conical tube/hybridization oven at 37°C with overhead shaking option (or equivalent)
70 or 100 µm Falcon® cell strainer (cat no. 431751/431752; Corning)
TSM base and TSM complete (for details see "Medium recipes")

Procedure:

1) Transfer the tumor sample to a cell culture dish (without PBS or medium) and mince thoroughly using sterile scalpels or scissors
2) Add PBS (-Ca/Mg) and transfer all tumor fragments to a 100 mL glass bottle and fill with PBS to a final volume of 20 to 100 mL (depending on the size of the sample).
3) Add 6 µl trypsin (10 mg/mL) per mL PBS (i.e., 120 µl to 20 mL PBS) and 40 µl collagenase II (50 mg/mL) per mL PBS (i.e., 800 µl to 20 mL PBS)
4) Incubate for 90 min at 37°C with permanent agitation on a magnetic stirrer (200 rpm)
5) Add 6 µl trypsin inhibitor (10 mg/mL) per mL PBS (i.e., 240 µl to 20 mL PBS) to stop the enzymatic digestion.
6) Prepare a 1:1 mix of 1 M MgCl2 and DNase (2 mg/mL) and repeatedly add 60 µl of the mix to the sample until the tumor fragments settle on the bottom of the bottle/tube; DNase is sensitive to mechanical stress (do not vortex, avoid excessive shear stress)
7) Filter through a 70 µm or 100 µm cell strainer; spin down (400-500 g, 10 min, RT)
8) Resuspend the cell pellet in 2 mL ACK Lysing buffer (or more, depending on the size of the pellet) to lyse red blood cells and incubate for 2-5 min at RT; wash the cells once with PBS and spin down (400-500 g, 5 min RT). Repeat this step if necessary (high number or residual red blood cells). Omit this step, if the material is very limited (small cell pellet) or if there is no red "ring" of erythrocytes visible.
9) Resuspend the cell pellet in TSM complete (5-10 mL), count and seed according to the desired use. We cultured the cells on Corning® CellBIND® 6-well Clear Multiple Well Plates (cat no. 3335, Corning) for two to seven days prior to seeding on drug plates and to start the long-term cultures.

Note 2.3: Brain tumors and brain metastases

Material:

Leibovitz-L15 (cat. no 11415049; Thermo Fisher Scientific)
Papain (cat no. P3125-100MG; Sigma–Aldrich; 16-40 units/mg protein, store at 4°C)
0.5 M EDTA
DNase (cat no. D4513, Sigma–Aldrich); 10.000 Units/mL in 0.15 M NaCl; store at -20°C
ACK (ammonium-chloride-potassium) lysing buffer (cat. no. A1049201; Thermo Fisher Scientific)
10 cm cell culture dish
50 mL conical tubes
Sterile scalpels; scissors
70 or 100 µm Falcon® cell strainer (cat no. 431751/431752; Corning)

TSM base and TSM complete (for details see “Medium recipes”)

Procedure:

1) Prepare papain dissociation medium:

- 5 mL Leibovitz-L15
- 60 Units papain (= 12 U/mL f.c.; vortex papain solution prior to pipetting)
- 5 µl 0.5 M EDTA (0.5 mM f.c.)
- Incubate the papain dissociation medium at 37°C for 15 min (until the solution becomes clear)
- Add 100 µl DNase (=200 U/mL f.c.); DNase is sensitive to mechanical stress (do not vortex, avoid excessive shear stress)

2) Transfer the tumor sample to a cell culture dish (without PBS or medium) and mince thoroughly using sterile scalpels or scissors
3) Add papain dissociation medium (+ DNase) and transfer all tumor fragments to a 50 mL conical tube.
4) Incubate for ~25 min at 37°C (water bath) and mix every 5-10 min by inverting the tube several times.
5) Filter through a 70 µm or 100 µm cell strainer; spin down (400-500 g, 10 min, RT)
6) Resuspend in 1 mL ACK Lysing buffer and incubate for 2 min at RT
7) Fill tube with TSM base and spin down cells (400-500 g, 10 min, RT)
8) Optional: repeat steps 6 and 7, if necessary (considerable red “ring” of erythrocytes in the pellet)

   Note of caution: Omit red blood cell lysis, if the material is very limited (small cell pellet), or if there is no red “ring” of erythrocytes visible.

9) Resuspend the cell pellet in TSM complete (5-10 mL), count and seed according to the desired use. We cultured the cells on Corning® CellBIND® 6-well Clear Multiple Well Plates (cat no. 3335, Corning) for two to seven days prior to seeding on drug plates and to start the long-term cultures.

Note 2.4: Neuroblastomas

Materials:

Neurobasal-A medium (cat. no. 10888022; Life Technologies)
Trypsin (cat no. T9935, Sigma–Aldrich; stock solution 10 mg/mL, store at -80°C)
Trypsin Inhibitor (cat. no. T6522, Sigma–Aldrich; stock solution 10 mg/mL, store at 4°C)

DNase (cat no. D4513, Sigma–Aldrich; stock solution 2 mg/mL in 0.15 M NaCl; store at -20°C)
1 M $MgCl_2$ (cat. no. AM9530G; Thermo Fisher Scientific)
ACK (ammonium-chloride-potassium) lysing buffer (cat. no. A1049201; Thermo Fisher Scientific)
10 cm cell culture dish
Sterile scalpels; scissors
50 mL conical tubes
70 or 100 µm Falcon® cell strainer (cat no. 431751/431752; Corning)
TSM base and TSM complete (for details see "Medium recipes")

Procedure:

1) Transfer the tumor sample to a cell culture dish (without medium) and mince thoroughly using sterile scalpels or scissors
2) Add Neurobasal-A medium, transfer all tumor fragments to a 50 mL conical tube, and fill with Neurobasal-A to a final volume of 5 to 50 mL (depending on the size of the sample).
3) Add 12 µl trypsin (10 mg/mL) per mL (i.e., 240 µl to 20 mL DMEM)
4) Incubate for 10 min at 37°C (incubator or waterbath)
5) Add 12 µl trypsin inhibitor (10 mg/mL) per mL (i.e., 240 µl to 20 mL DMEM) to stop the enzymatic digestion.
6) Prepare a 1:1 mix of 1 M $MgCl_2$ and DNase (2 mg/mL) and repeatedly add 60 µl of the mix to the sample until the tumor fragments settle on the bottom of the tube.
7) Filter through a 70- or 100 µm cell strainer; spin down (400-500 g, 10 min, RT)
8) Resuspend the cell pellet in 2 mL ACK Lysing buffer (or more, depending on the size of the pellet) to lyse red blood cells and incubate for 2-5 min at RT; fill up to 10 mL with TSM base and spin down the cells (400-500 g, 5 min RT). Omit this step, if the material is very limited (small cell pellet), or if there is no red "ring" of erythrocytes visible.
9) Optional: repeat step 8, if necessary (considerable red "ring" of erythrocytes in the pellet)
10) Resuspend the cell pellet in TSM complete (5-10 mL), count and seed according to the desired use. We cultured the cells on Corning® CellBIND® 6-well Clear Multiple Well Plates (cat no. 3335, Corning) for two to seven days prior to seeding on drug plates and to start the long-term cultures.

Note 3: Primary cell culture

Materials:

TSM base and TSM complete (see “Medium recipes”)
TrypLE Express (cat no. 12604-013; Gibco)
Corning® CellBIND® 6-well Clear Multiple Well Plates (cat no. 3335, Corning)
T-25, T-75 and T-175 cell culture flasks for suspension cells (cat nos. 833.910.502, 833.911.502 and 833.912.502; Sarstedt)
Synth-a-Freeze (cat no. A12542-01, Gibco)

Dissociation for preparation of single-cell solutions (drug screens) or subculturing:

Note: Primary cells like to grow very dense, avoid splitting them to early. Subculture, when an average spheroid diameter of approx. 700-1000 nm is reached.
Recommended splitting ratio: 1:2 –1:5.

1) Transfer medium with spheroids and free floating cells to a 50 mL conical tube and spin down (400-500 g, 5 min, RT)
2) Add 1-5 mL TrypLE to the spheroid pellet (depending on the pellet size)
3) Dissociate the spheroids by pipetting up and down 20-30x until a single-cell suspension is obtained. Incubate at 37°C for a maximum of 5 min if the cells do not easily dissociate; avoid generating air bubbles. Check the dissociation of the spheroids under the microscope
4) If present, detach adherent cells by adding TrypLE express (i.e., 1 mL per well in a 6-well plate), incubate at 37°C for a maximum of 5 min; check detachment under a microscope.
5) Combine cells from 2) and 3) by adding 4-5 mL TSM base; pellet cells at 400-500 g (5-10 min, RT)
6) Resuspend in TSM complete, count and seed in desired format

Freezing:

1) Transfer all spheroids and detached adherent cells to a 15- or 50 mL conical tube
2) Spin down at 400-500 × g for 5 min
3) Discard the supernatant and resuspend the spheroids and cells in the respective volume of Synth-A-Freeze (1 mL per cryotube).
    - 1 mL Synth-a-Freeze for cells from 6-well format
    - 1 mL Synth-a-Freeze for cells from T-25
    - 3 mL Synth-a-Freeze for cells from T-75
    - 5 mL Synth-a-Freeze for cells from T-175

4) Slowly freeze down to -80°C overnight (e.g., using a Mr. Frosty™ Freezing Container; cat no. 5100-0001 Thermo Fisher Scientific), and transfer cryovials to liquid nitrogen storage as soon as possible.

Thawing:

1) Prepare a T25 suspension flask with 6 mL TSM complete
2) Quickly thaw the cryovial with the warmth of your hand or in a 37°C waterbath until a small ice crystal is left only
3) Transfer the thawed cell suspension (1 mL volume) to the T25 suspension flask with TSM complete and mix thoroughly (avoid air bubbles)
   Note: We do not spin down the cells after thawing, as we observed that avoiding centrifugation increases cell viability.
4) Either add 1 mL TSM complete or exchange half of the medium every two to three days, or subculture the cells, if appropriate

Note 4: Medium recipes

Tumor Stem Medium (TSM) base

Reagent	Vendor	Catalog no.	Volume
Neurobasal-A Medium (1X), liquid	Thermo Fisher	21103049	250 mL
D-MEM/F-12 (1X), liquid, 1:1	Thermo Fisher	11330-032	250 mL
HEPES Buffer Solution (1 M)	Thermo Fisher	15630-080	5 mL
MEM Sodium Pyruvate Solution 100 mM (100X), liquid	Thermo Fisher	11360-070	5 mL
MEM Non-Essential Amino Acids Solution 10 mM (100X), liquid	Thermo Fisher	11140-050	5 mL
L-GLUTAMINE SOLUTION BIOXTRA, 200 MM, SO	Sigma–Aldrich	G7513-100ML	5 mL
PENICILLIN -STREPTOMYCIN SOLUTION*STABIL	Sigma–Aldrich	P4333-100ML	5 mL

Filter TSM base (Stericup-GP 500 m Express Plus PES.22 µm, Merck, Catalog #SCGPU05RE) and store 50 mL aliquots at 4°C (if you want to keep the TSM base for longer than one to two weeks, add L-glutamine and pen/strep to TSM complete).

TSM complete (base medium + supplements; store one to max. two weeks at 4°C)

Reagent	Vendor	Catalog no.	Working conc.	Stock conc.	Volume
TSM Base	--	--	--	--	50 mL

B-27 Supplement Minus Vitamin A (50X), liquid	Thermo Fisher	12587-010	--	--	1 mL
H-EGF	Peprotech	AF-100-15B	20 ng/mL	100 µg/mL	10 µL
H-FGF-basic-154	Peprotech	100-18B	20 ng/mL	100 µg/mL	10 µL
H-PDGF-AA	Peprotech	100-13A	10 ng/mL	20 ug/mL	25 µL
Heparin Solution, 0.2%	StemCell Technologies, Inc.	H3149-10KU	2 µg/mL	2 mg/mL	50 L

Note 5: Drug screening and metabolic activity assay

Materials

VI-Cell XR Reagent Pack, cat no. 383260; Beckman Coulter), including trypan blue, to assess cell viability by trypan blue exclusion
Disposable counting chamber slides (cat no. CHT4-SD100; Nexcelom)
Acridine orange/propidium iodide (AO/PI; cat no. CS2-0106; Nexcelom) to count total and viable cell numbers (for Cellometer K2)
10 mL sterile reservoirs (cat no. 4332; Integra Biosciences)
384-well black/clear round bottom ultra-low attachment spheroid microplates (cat no. 3830; Corning)
Cell Titer Glo 2.0 (Promega)

Instruments

Vicell XR (Beckmann Coulter)
Cellometer K2 Fluorescent Cell Counter (Nexcelom)
Picus 8-ch 10-300 µl electronic pipette (Sartorius)
FLUOstar OPTIMA Microplate Reader (BMG LABTECH)
PHERAstar FS Multimode Microplate Reader (BMG LABTECH)
Spark Multimode Microplate Reader (TECAN)

Seeding of cells on drug plates:

1. Prepare single-cell solution in TSM complete from patient-derived cell cultures as described in “Primary cell culture”
2. Determine the number of viable cells and prepare a master cell mix calculating (if possible) 1000 cells and 25 µl TSM complete per well of a 384-well plate.
3. Spin down preprinted assay plates for 3 min at 300 g
4. Dispense 25 µl/well of the cell master mix on the assay plates

5. Spin down assay plates with dispensed cells (300 × g for 3-5 min) to allow cell aggregation and spheroid formation
6. Incubate the plates at 37°C/5% CO_2 for 72 h.

Metabolic activity readout

1. Adapt assay plates to room temperature for approximately 10 min
2. Add 15 µl (25 µl if plates were also used for imaging experiments) CTG 2.0 per well (to 25 µl medium with cells, 40 µl or 50 µl total volume, respectively)
3. Shake for 5 min on a plate shaker (400 rpm; protected from light)
4. Spin down plates for 3 min at 300 g
5. Further incubate for 20 minutes (protected from light) at room temperature.
6. Perform luminescence readout (luminescence mode, bottom optic, integration time 1000 ms), e.g., with FLUOstar OPTIMA, or, preferably, with the more sensitive high-throughput PHERAstar FS(X) or TECAN Spark multimode plate readers

Supplementary References

1 Eisemann, T. *et al.* Podoplanin expression is a prognostic biomarker but may be dispensable for the malignancy of glioblastoma. *Neuro-oncology* **21**, 326-336, doi:10.1093/neuonc/noy184 (2019).

2 Stewart, E. *et al.* Orthotopic patient-derived xenografts of paediatric solid tumours. *Nature* **549**, 96-100, doi:10.1038/nature23647 (2017).

3 Kodack, D. P. *et al.* Primary Patient-Derived Cancer Cells and Their Potential for Personalized Cancer Patient Care. *Cell reports* **21**, 3298-3309, doi:10.1016/j.celrep.2017.11.051 (2017).

4 Pauli, C. *et al.* Personalized In Vitro and In Vivo Cancer Models to Guide Precision Medicine. *Cancer discovery* **7**, 462-477, doi:10.1158/2159-8290.CD-16-1154 (2017).

5 Lin, G. L. & Monje, M. A Protocol for Rapid Post-mortem Cell Culture of Diffuse Intrinsic Pontine Glioma (DIPG). *J Vis Exp*, doi:10.3791/55360 (2017).
