# Supplementary material for: Drug sensitivity profiling of 3D tumor tissue cultures in the pediatric precision oncology program INFORM
Source: NPJ Precis Oncol. 2022 Dec 27;6:94. doi: 10.1038/s41698-022-00335-y (PMC9794727; doi:10.1038/s41698-022-00335-y)
Supplement: Supplementary file 3 — REPORTING SUMMARY [file 41698_2022_335_MOESM3_ESM.pdf]

nature portfolio

Corresponding author(s): Ina Oehme

Last updated by author(s): 31. Oct 2022

Reporting Summary

Nature Portfolio wishes to improve the reproducibility of the work that we publish. This form provides structure for consistency and transparency in reporting. For further information on Nature Portfolio policies, see our Editorial Policies and the Editorial Policy Checklist.

Statistics

For all statistical analyses, confirm that the following items are present in the figure legend, table legend, main text, or Methods section.

n/a	Confirmed	
☐	☒	The exact sample size (*n*) for each experimental group/condition, given as a discrete number and unit of measurement
☐	☒	A statement on whether measurements were taken from distinct samples or whether the same sample was measured repeatedly
☐	☒	The statistical test(s) used AND whether they are one- or two-sided<br>*Only common tests should be described solely by name; describe more complex techniques in the Methods section.*
☒	☐	A description of all covariates tested
☐	☒	A description of any assumptions or corrections, such as tests of normality and adjustment for multiple comparisons
☐	☒	A full description of the statistical parameters including central tendency (e.g. means) or other basic estimates (e.g. regression coefficient) AND variation (e.g. standard deviation) or associated estimates of uncertainty (e.g. confidence intervals)
☒	☐	For null hypothesis testing, the test statistic (e.g. *F*, *t*, *r*) with confidence intervals, effect sizes, degrees of freedom and *P* value noted<br>*Give P values as exact values whenever suitable.*
☒	☐	For Bayesian analysis, information on the choice of priors and Markov chain Monte Carlo settings
☒	☐	For hierarchical and complex designs, identification of the appropriate level for tests and full reporting of outcomes
☐	☒	Estimates of effect sizes (e.g. Cohen's *d*, Pearson's *r*), indicating how they were calculated

Our web collection on statistics for biologists contains articles on many of the points above.

Software and code

Policy information about availability of computer code

Data collection	Codes are available on GitHub: https://github.com/iTReX-Shiny/iTReX; https://github.com/KiTZ-Heidelberg/INFORM-TDSU-Manuscript.
Data analysis	An extended (including Cmax values) version of iTReX was used for the manuscript to analysis metabolic data obtained from the drug screens. The second code listed above was used for automated QC check of the screened plates.

For manuscripts utilizing custom algorithms or software that are central to the research but not yet described in published literature, software must be made available to editors and reviewers. We strongly encourage code deposition in a community repository (e.g. GitHub). See the Nature Portfolio guidelines for submitting code & software for further information.

Data

Policy information about availability of data

All manuscripts must include a data availability statement. This statement should provide the following information, where applicable:

- Accession codes, unique identifiers, or web links for publicly available datasets
- A description of any restrictions on data availability
- For clinical datasets or third party data, please ensure that the statement adheres to our policy

Data are available as supplemental material and upon request. Detailed SOPs are available as Supplementary Notes.
The INFORM patient expression data are not publicly available (patient data protection).
WES, lcWGS, RNA-sequencing, and methylation data generated by this study are available from the European Genome Archive, accession number

EGAS00001005112. EGA requires controlled access, there is a corresponding Data Access Committee (DAC) to determine access permissions. Access to actual data files is not managed by the EGA.

Human research participants

Policy information about studies involving human research participants and Sex and Gender in Research.

Reporting on sex and gender	Information on sex (biological attribute) is collected in the eCRF. No information on gender was collected of this pediatric patients.
Population characteristics	See section „Patients and Baseline Characteristics" in Cancer Discov (2021) 11 (11): 2764–2779.
Recruitment	See sections "Study Design, Eligibility, and Patients" AND "Procedures" in Cancer Discov (2021) 11 (11): 2764–2779.
Ethics oversight	The study was conducted in accordance with Good Clinical Practice guidelines and the Declaration of Helsinki. All patients, their legally acceptable representatives, or both (if possible) provided written informed consent. Approval for the study protocol (and any modifications thereof) was obtained from independent ethics committees and the institutional review board at each participating center. The study was registered with the German Clinical Trial Register, number DRKS00007623.

Note that full information on the approval of the study protocol must also be provided in the manuscript.

Field-specific reporting

Please select the one below that is the best fit for your research. If you are not sure, read the appropriate sections before making your selection.

☒ Life sciences ☐ Behavioural & social sciences ☐ Ecological, evolutionary & environmental sciences

For a reference copy of the document with all sections, see nature.com/documents/nr-reporting-summary-flat.pdf

Life sciences study design

All studies must disclose on these points even when the disclosure is negative.

Sample size	n=132
Data exclusions	QC check; data not fullfilling the QC criteria were excluded (all listed in Suppl. Table 1)
Replication	DSP of limited patient material was performed in technical duplicates.
Randomization	n/a - samples were screened when ready, without any systematic order
Blinding	all screens were run blinded as molecular data was not available during DSP

Reporting for specific materials, systems and methods

We require information from authors about some types of materials, experimental systems and methods used in many studies. Here, indicate whether each material, system or method listed is relevant to your study. If you are not sure if a list item applies to your research, read the appropriate section before selecting a response.

Materials & experimental systems

n/a	Involved in the study	
☒	☐	Antibodies
☒	☐	Eukaryotic cell lines
☒	☐	Palaeontology and archaeology
☒	☐	Animals and other organisms
☒	☐	Clinical data
☒	☐	Dual use research of concern

Methods

n/a	Involved in the study	
☒	☐	ChIP-seq
☒	☐	Flow cytometry
☒	☐	MRI-based neuroimaging
